# cAMP-dependent cell differentiation triggered by activated CRHR1 in hippocampal neuronal cells

**DOI:** 10.1038/s41598-017-02021-7

**Published:** 2017-05-16

**Authors:** Carolina Inda, Juan José Bonfiglio, Paula A. dos Santos Claro, Sergio A. Senin, Natalia G. Armando, Jan M. Deussing, Susana Silberstein

**Affiliations:** 1 Instituto de Investigación en Biomedicina de Buenos Aires (IBioBA)-CONICET-Partner Institute of the Max Planck Society, Buenos Aires, Argentina; 2 DFBMC, Facultad de Ciencias Exactas y Naturales, Universidad de Buenos Aires, Buenos Aires, Argentina; 3 Max Planck Institute of Psychiatry, Department of Stress Neurobiology and Neurogenetics, Molecular Neurogenetics, Munich, Germany; 4 Max Planck Institute for Biology of Ageing, Cologne, Germany

## Abstract

Corticotropin-releasing hormone receptor 1 (CRHR1) activates the atypical soluble adenylyl cyclase (sAC) in addition to transmembrane adenylyl cyclases (tmACs). Both cAMP sources were shown to be required for the phosphorylation of ERK1/2 triggered by activated G protein coupled receptor (GPCR) CRHR1 in neuronal and neuroendocrine contexts. Here, we show that activated CRHR1 promotes growth arrest and neurite elongation in neuronal hippocampal cells (HT22-CRHR1 cells). By characterising CRHR1 signalling mechanisms involved in the neuritogenic effect, we demonstrate that neurite outgrowth in HT22-CRHR1 cells takes place by a sAC-dependent, ERK1/2-independent signalling cascade. Both tmACs and sAC are involved in corticotropin-releasing hormone (CRH)-mediated CREB phosphorylation and *c-fos* induction, but only sAC-generated cAMP pools are critical for the neuritogenic effect of CRH, further highlighting the engagement of two sources of cAMP downstream of the activation of a GPCR, and reinforcing the notion that restricted cAMP microdomains may regulate independent cellular processes.

## Introduction

The second messenger adenosine 3′-5′-cyclic monophosphate (cAMP) is involved in multiple signalling mechanisms activated in response to extracellular signals, which in turn regulate numerous cellular functions. A critical role of cAMP in cell differentiation and proliferation has been demonstrated and, paradoxically, cAMP is able to promote opposite effects depending on the involved cell type^1^. In the central nervous system, cAMP enhances neuronal differentiation and is involved in many neuronal processes that include regulation of synaptic plasticity, memory formation and cell survival in both the developing and adult brain.

It was first demonstrated in cultured dorsal root ganglia from chick embryos that elevated cAMP enhanced axon elongation^2^. Over the years, a wealth of studies has explored the key role of cAMP in the growth and guidance of axons, and it has been established that intracellular levels of cAMP are related to the neuritogenic capacity of neurons^3, 4^.

G protein-coupled receptor (GPCR) stimulation is the best-characterised signalling event that leads to increased intracellular cAMP levels. GPCRs couple the binding of ligands, such as hormones or neuropeptides, to the stimulation of heterotrimeric G proteins, which regulate transmembrane adenylyl cyclase (tmACs) activity^5^.

The corticotropin-releasing hormone receptor 1 (CRHR1) is a critical regulator of the neuroendocrine, behavioural and autonomic stress response. Accumulating evidence showed that dysregulation of the CRHR1 system is causally linked to the onset of mood and anxiety disorders^6, 7^. CRHR1 belongs to the class B/secretin-like GPCR family and preferentially signals via Gαs coupling, resulting in the activation of the tmACs and increased cAMP levels^8^. We have recently reported that CRHR1-mediated cAMP production does not only depend on G protein-dependent tmAC activation, but that it also involves an atypical source of cAMP, the G protein-independent soluble adenylyl cyclase (sAC). Remarkably, we found that CRHR1 continues to generate cAMP after internalization and that sAC is essential for this process whereas tmACs are not^9^. These findings are in line with the emerging appreciation of the importance of spatio-temporal resolution in signalling mechanisms^10^.

Neuronal differentiation is achieved by complex cellular processes, which include morphological changes and growth arrest in addition to biochemical changes, increased electrical excitability and specific gene expression programmes. The use of cellular models, such as the neuroendrocrine cell line PC12, derived from a rat phaeochromocytoma, has not only been useful to investigate the mechanisms involved in neurite elongation, but also to assess how signalling pathways integrate extracellular signals to promote common or distinct biological outcomes^11^. For example, it has been well demonstrated that neurite outgrowth in PC12 cells can be achieved by receptor tyrosine kinase (RTK)-activating neurotrophins, such as nerve growth factor (NGF), or neuropeptides that elevate intracellular cAMP via GPCR-activation, such as pituitary adenylate cyclase–activating polypeptide (PACAP). Common to these signalling cascades is a sustained ERK1/2 activation, critical for neuritogenesis. In contrast, a transient phosphorylation of ERK1/2, elicited in response to epidermal growth factor (EGF) for example, leads to cell proliferation in PC12 cells. Although a cAMP-dependent ERK1/2 activation seems to be a general characteristic of neuronal and endocrine cells^12^, whether ERK1/2 is critical for neurite outgrowth may depend on the particular cell context.

We used the mouse hippocampal cell line HT22 as a cellular model to study the signalling pathways activated by CRHR1. We have previously characterised the mechanisms involved in cAMP production and ERK1/2 activation upon CRH stimulation^9, 13^. Having observed that upon CRH addition HT22 cells stably expressing CRHR1 (HT22-CRHR1) undergo morphological changes, in this work we explored the molecular components critical for this effect in order to further understand the integration and crosstalk among the different signalling cascades downstream the GPCR CRHR1.

## Results

### CRHR1 activation elicits a sustained cAMP response in primary cultured neurons and HT22-CRHR1 cells

**Figure 1:**
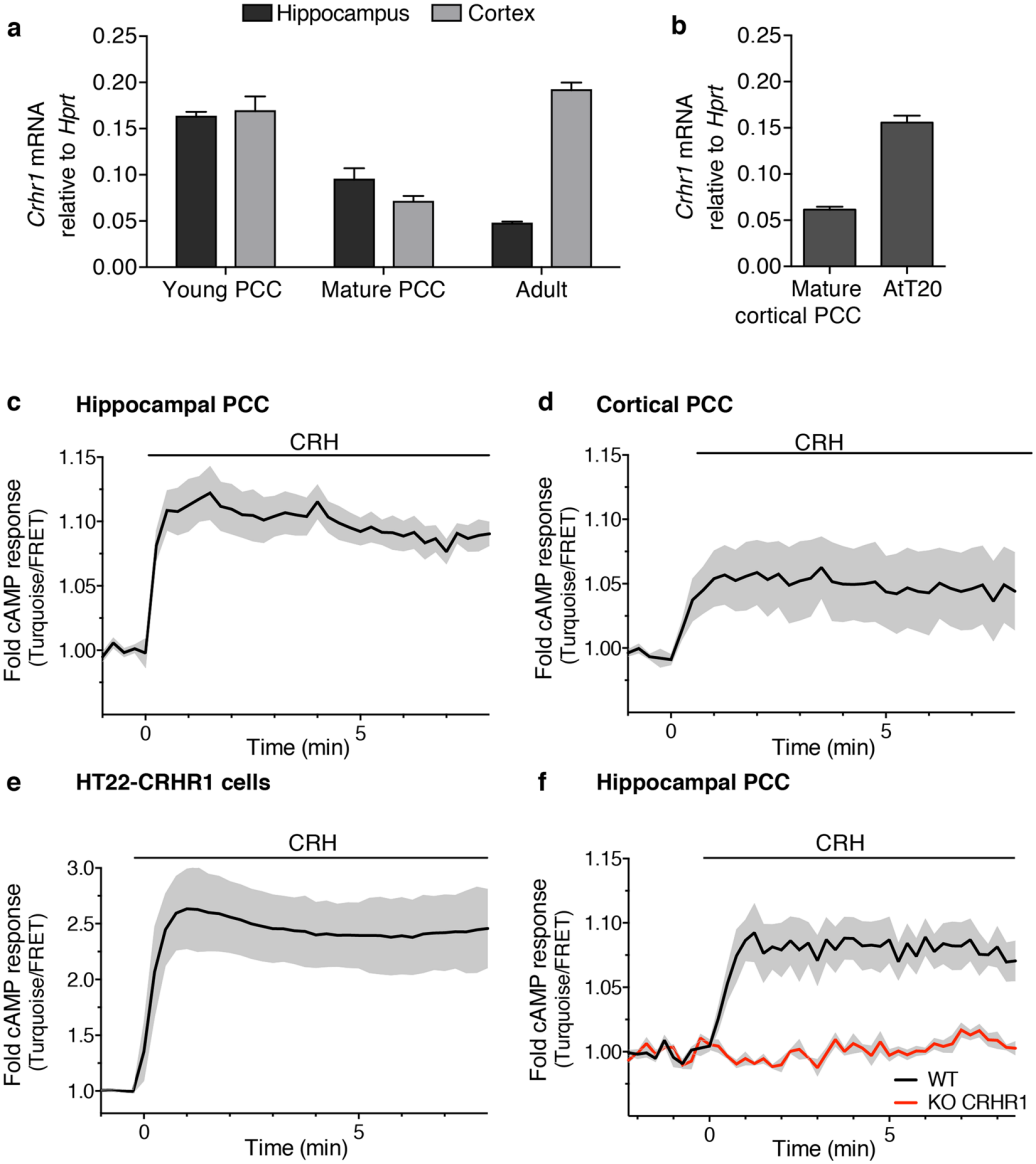
cAMP response of CRH-activated CRHR1 in neurons. Expression of *Crhr1* was assessed by RT-q-PCR. (**a**) mRNA levels of *Crhr1* were analysed in young (DIV 5) and mature (DIV 12) primary hippocampal or cortical cell cultures (PCC) and in the adult brain structures. (**b**) mRNA levels of *Crhr1* were analysed in mature cortical PCC and AtT20 cell line. *Crhr1* mRNA levels were normalized to *Hprt* (mean ± SEM, n = 3). (**c–f**) Time course of FRET changes were measured in single cells transfected with Epac-S^H187^ construct. Primary hippocampal (**c**,**f**) or cortical (**d**) neurons as well as HT22-CRHR1 cells (**e**) were analysed. (**f**) Primary cultures were derived from conditional KO mice lacking CRHR1 in glutamatergic neurons. The cAMP response to CRH in WT and KO neurons was analysed in a mixed population in the same microscope field (KO neurons express tdTomato). Cells were stimulated at time 0 with CRH (**c,d,f**, 100 nM; **e**, 10 nM). Traces are representative of three independent experiments (mean ± SEM, 20–25 cells).

We have previously determined that CRH stimulation of CRHR1 leads to a rapid and sustained increase of intracellular cAMP levels using the HT22-CRHR1 cell line as a neuronal hippocampal model^9^. Here, we asked whether a prolonged cAMP production was also characteristic of the CRH response in primary neurons. We first detected *Crhr1* mRNA by quantitative real-time PCR (q-RT-PCR) in embryonic primary neuronal cultures prepared from hippocampus and cortex (Fig. 1a) in line with previous reports^7, 14, 15^. *Crhr1* mRNA was detected in the same structures in the adult mouse brain (Fig. 1a) and in the corticotroph-derived cell line AtT20 as well (Fig. 1b).

We measured the cAMP response elicited by CRH in neurons at the single-cell level in real time using the FRET-based biosensor Epac-S^H187^ ^16^. In both hippocampal and cortical primary cell cultures, upon bath application of CRH, FRET responses were decreased evidencing an increase in the cellular cAMP levels (Fig. 1c,d). Remarkably, cAMP levels stayed elevated for at least 10 min after CRH addition, recapitulating the sustained cAMP response observed in HT22-CRHR1 cells (Fig. 1e). We verified that CRH addition produced a decrease of acceptor emission (cp173Venus) and a corresponding increase in donor emission (mTurquoise2), confirming that the observed changes were caused by a FRET reduction (Supplementary Fig. 1a,c). The addition of forskolin after CRH stimulation further decreased FRET levels, indicating that the probes were not saturated (Supplementary Fig. 1b,d).

We prepared hippocampal primary cell cultures using conditional CRHR1 knockout mice lacking CRHR1 in glutamatergic forebrain neurons (*CRHR1* ^*CKO-Glu*^) bred to tdTomato reporter mice (Ai9; *R26* ^*CAG::LSLtdTomato*^). In these primary cultures CRHR1 is selectively deleted in glutamatergic neurons as visualized by simultaneous activation of tdTomato^7, 17^. We transfected neurons with Epac-S^H187^ and measured the cAMP levels in response to CRH in the mixed population of wild-type neurons and CRHR1-deficient neurons expressing tdTomato in the same microscope field. While rapid and sustained cAMP levels were observed in the wild-type neurons, no response was detected in neurons lacking CRHR1 (Fig. 1f), confirming that the FRET measurement was a specific detection of cAMP and that the cAMP response was fully dependent on CRHR1. This is in line with no CRHR2 expression detected in these primary neurons^14^. These results indicate that the cAMP response triggered by CRH-activated CRHR1 in neurons and in HT22-CRHR1 cells follow a similar profile, validating the use of HT22-CRHR1 cells^9, 13^ as a reliable cellular model to study CRHR1 signalling.

### CRHR1 activation promotes fast neuronal differentiation in HT22-CRHR1 cells

**Figure 2:**
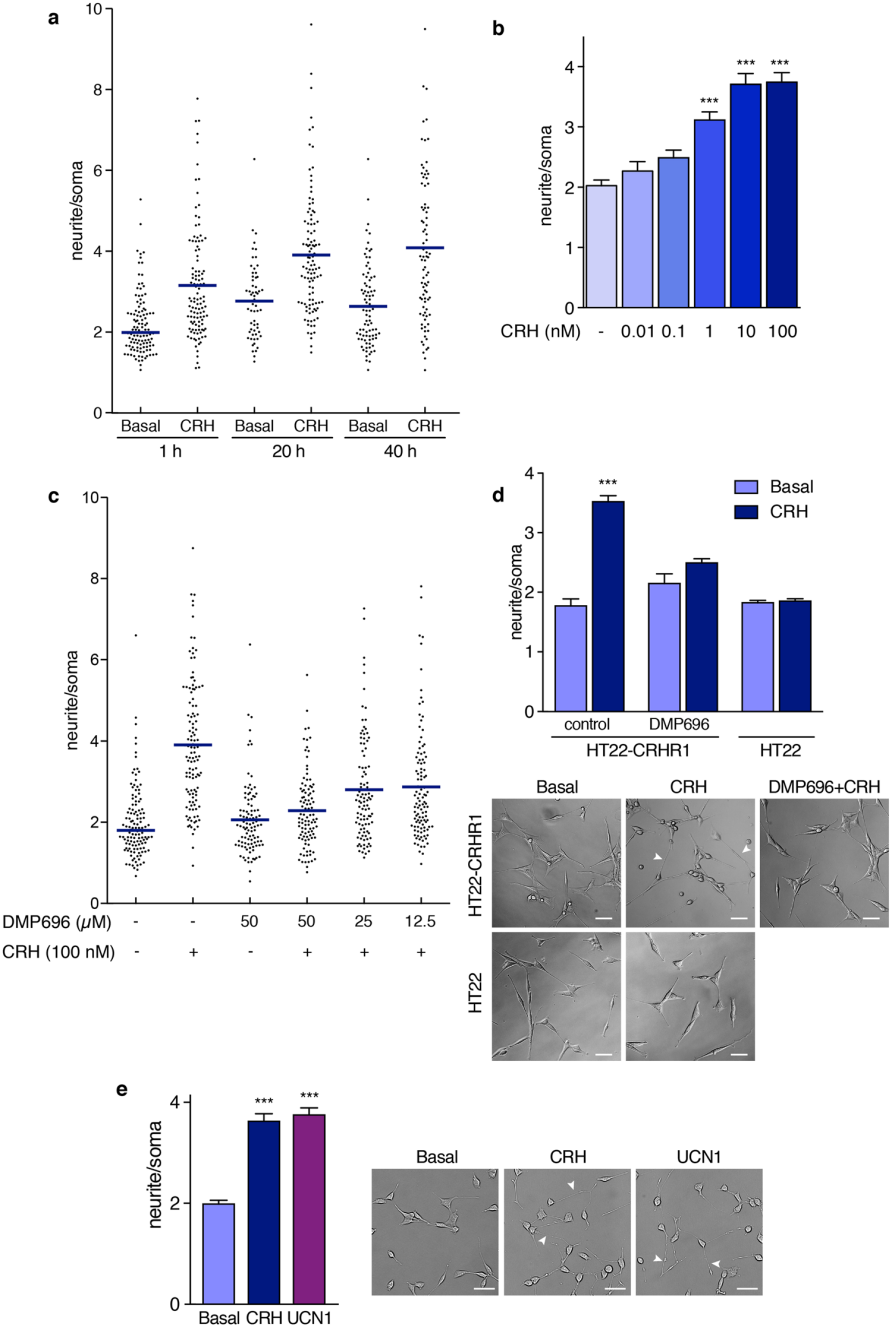
CRHR1 activation promotes neurite outgrowth in HT22-CRHR1 cells. (**a**) HT22-CRHR1 cells were stimulated with 100 nM CRH and neurite outgrowth was determined in individual cells at the indicated time points as the ratio between the longest neurite and the soma in each cell, showing the median by horizontal lines. (**b**) CRH-neuritogenic effect in HT22-CRHR1 cells after 20 h-treatment was evaluated in presence of vehicle or different concentrations of CRH in OptiMEM. Data: mean ± SEM (n = 3). ***p < 0.001 respect to basal by one-way ANOVA followed by Tukey post test. (**c**) HT22-CRHR1 cells were stimulated with 100 nM CRH in presence of different concentrations of DMP696, an specific CRHR1 antagonist. Neurite outgrowth was determined in individual cells after 20 h-treatment, indicating the median by horizontal lines. (**d**,**e**) Quantification of CRH or UCN1-mediated neuritogenic effect in HT22 or HT22-CRHR1 cells in presence or absence of DMP696 (50 µM). Data: mean ± SEM, n = 3. ***p < 0.001 respect to basal by repeated measures one-way ANOVA followed by Tukey post test. A representative photograph is shown for each treatment. Arrowheads point to neurite extensions. Scale bars, 50 μm.

When cultured in presence of serum, HT22-CRHR1 cells show a flattened, spindle-shaped morphology. We observed that CRH stimulation triggered a fast morphological change in HT22-CRHR1 cells, characterised by neurite elongation and a more rounded soma (Supplementary Video 1–2 and Fig. 2a–d). Although HT22-CRHR1 are multipolar cells, in general one of the processes was the most elongated upon CRH addition. Thus, we decided to quantify the morphological change as the ratio between the length of the longest neurite and the soma diameter. Compared to the unstimulated control, CRH augmented the proportion of cells with longer neurites in the population (Fig. 2a). This effect was evident 1 h after CRH addition, but it was emphasized at longer times (24 h and 48 h after treatment). Serum deprivation induced a subtle morphological cell change (compare basal 1 h vs 24 and 48 h) but a strong CRH-dependent neuritogenic effect was significant at CRH concentrations as low as 1 nM (Fig. 2b).

Pre-incubation with a specific CRHR1 antagonist, DMP696, prevented the neurite outgrowth upon CRH stimulation in a concentration-dependent manner (Fig. 2c,d). HT22-CRHR1 cells do not express CRHR2^13^ and CRH did not induce morphological changes in the HT22 parental cell line (Fig. 2d), suggesting that the effect of CRH is via the activation of CRHR1.

CRH is not the only endogenous ligand for CRHR1; the urocortins -UCN1, UCN2 and UCN3- are CRH-related peptides also involved in the stress response^15, 18^. Whereas UCN2 and UCN3 are highly selective CRHR2 ligands, UCN1 binds to both CRHR1 and CRHR2^19, 20^. To examine whether this neuritogenic effect depended on a particular CRHR1 typical ligand, we compared the neurite outgrowth elicited by CRH and UCN1 without detecting significant differences between stimuli (Fig. 2e and Supplementary Video 3). Taken together, these results indicate that CRHR1 activation mediates the neurite outgrowth in HT22-CRHR1 cells.

**Figure 3:**
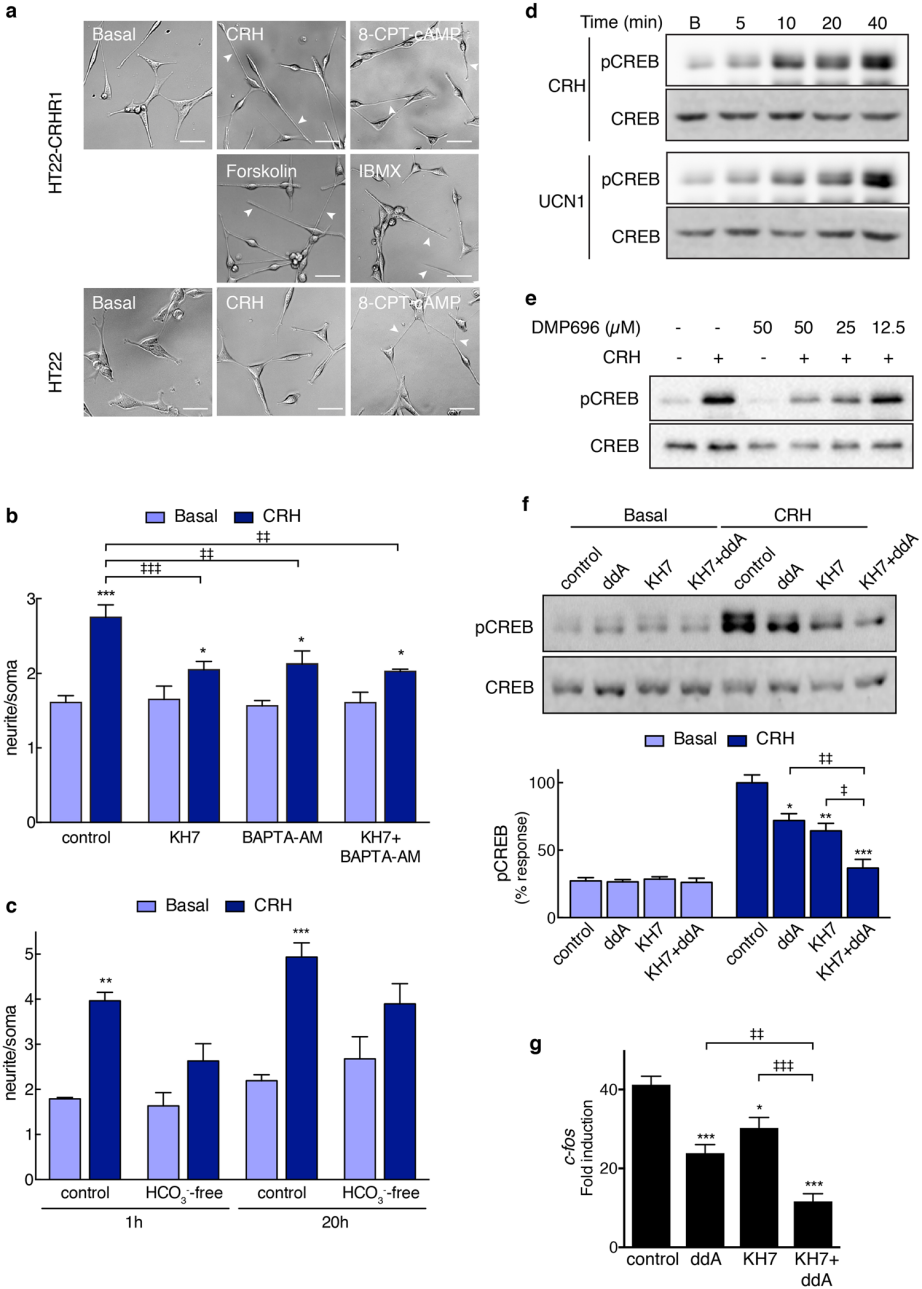
HT22-CRHR1 cell differentiation depends on cAMP and activated sAC. (**a**) HT22 or HT22-CRHR1 cells were stimulated with 100 nM CRH, 50 µM cell-permeable cAMP analog 8-CPT-cAMP, 50 µM tmAC activator forskolin or 500 µM phosphodiesterase inhibitor IBMX. A representative photograph is shown for each treatment. Arrowheads point to neurite extensions. Scale bars, 50 μm. (**b**) Neurite outgrowth was determined in HT22-CRHR1 cells stimulated with 100 nM CRH for 2 h in presence of vehicle (control), sAC-specific inhibitor (7.5 µM KH7), or calcium chelator (**c**, 5 µM BAPTA-AM). Data: mean ± SEM, n = 3. *p < 0.05, ***p < 0.001 respect to basal; ^‡^p < 0.05, ^‡‡^p < 0.01, ^‡‡‡^p < 0.001 between indicated treatments by one-way ANOVA followed by Tukey post test. (**c**) Neurite outgrowth was determined in HT22-CRHR1 cells stimulated with 100 nM CRH in HCO_3_^−^-free or 25 mM HCO_3_^−^ DMEM for the indicated time points. Data: mean ± SEM, n = 4. **p < 0.01, ***p < 0.001 respect to basal; by one-way ANOVA followed by Tukey post test. (**d**) HT22-CRHR1 cells were stimulated with 100 nM CRH or UCN1 at the indicated time points. (**e**) HT22-CRHR1 cells were stimulated for 40 min with 100 nM CRH in presence of different concentrations of CRHR1 antagonist DMP696. (**f,g**) HT22-CRHR1 cells were preincubated with vehicle (control) or sAC-specific (7.5 µM KH7) or tmAC-specific (50 µM ddA) inhibitors and stimulated with 100 nM CRH. (**e**,**f**) phosphorylated CREB (pCREB) and total CREB were determined by Western blot in 40-min cell lysates. (**g**) *c-fos* mRNA levels after 1 h were determined by RT-q-PCR and normalized to *Hprt*. Data: mean ± SEM, n = 3. *p < 0.05, ***p < 0.001 respect to control; ^‡^p < 0.05, ^‡‡^p < 0.01, ^‡‡‡^p < 0.001 between indicated treatments by one-way ANOVA followed by Tukey post test.

Numerous reports suggest that cAMP has a key role in the neurite elongation in response to GPCR ligands. We observed a morphological change similar to the one elicited by CRH when HT22-CRHR1 cells were incubated with 8-CPT-cAMP, a cell-permeable analogue of cAMP or compounds that increase intracellular cAMP levels, forskolin –by activation of tmACs- and IBMX –by PDEs inhibition- (Fig. 3a). Moreover, when we stimulated HT22-CRHR1 cells with isoproterenol, an agonist of β-adrenergic receptors which elicits a cAMP response^9^, we also observed neurite outgrowth (Supplementary Fig. 2a). Collectively, these results indicate that a rise in cAMP in the HT22 cell line leads to morphological changes characterised by the elongation of neurites. On the other hand, when we stimulated with CRH other cell lines, such as corticotroph-derived AtT20 (which endogenously express CRHR1) or fibroblast-derived 3T3L1 stably expressing CRHR1 (3T3L1-CRHR1), no significant morphological changes were observed (Supplementary Fig. 2b) although we have previously shown that CRH triggers a cAMP response in both cell sytems^9^. cAMP elevation by forskolin treatment did not cause neurite outgrowth in these cell lines either (Supplementary Fig. 2b), showing that a cAMP rise leads to neuritogenesis depending on specific properties of the cell type.

### sAC-generated cAMP is critical for the neuritogenic effect of CRH

We have recently demonstrated that, in addition to tmACs, sAC contributes to the CRH-activated CRHR1 cAMP response^9^. sAC is present in a wide variety of tissues, including neurons in the hippocampus, cortex, cerebellum, dorsal root ganglion (DRG) and spinal cord^21–24^. RT-PCR results confirmed that *sAC* mRNA was present in primary cultures from cortex and hippocampus as well as these structures in the adult brain (Supplementary Fig. 3).

The CRH-dependent morphological change was not affected in cells pre-incubated with tmAC-specific inhibitors^25^ (Supplementary Fig. 4a,b) but was blocked with sAC-specific inhibitors^25^ KH7 (Fig. 3b) and 2-HE (Supplementary Fig. 4c), confirming that sAC-generated cAMP pool was essential for neuritogenesis^9^. As a control, we verified that sAC inhibitor KH7 had no effect in forskolin-mediated neurite outgrowth (Supplementary Fig. 4d). These findings provide further evidence on the critical role of cAMP in cell morphological changes, and support the notion that different cAMP pools can be involved in different signalling mechanisms downstream the activated CRHR1.

We have previously demonstrated that cAMP production by CRHR1 largely depends on endocytosis, as the inhibition of receptor internalization diminishes CRH-triggered cAMP response in HT22-CRHR1 cells. Importantly, sAC, but not tmAC, is essential for endocytosis-dependent cAMP production^9^. We asked whether the endocytosis-dependent cAMP pool was critical for the CRH neuritogenic effect. Cells expressing a dominant-negative mutant of dynamin (DynK44A), which blocks CRHR1 internalization upon ligand stimulation^9, 13^, showed a slight decrease in the CRH-triggered neurite outgrowth (Supplementary Fig. 4e). This is consistent with a role of endocytosis contributing to the cAMP response dependent on activated CRHR1^9^. However, considering the different impact of blocking sAC directly (Fig. 3b, Supplementary Fig. 4c) or blocking endocytosis (Supplementary Fig. 4e) in CRH-mediated neuritogenesis, our results strengthen the concept of a function of sAC not restricted to an endosome-based mechanism of cAMP production, being also playing a role in the acute generation of cAMP that is involved in the early phase of ERK1/2 activation^9^.

sAC is insensitive to G protein regulation, but is directly activated by calcium^26, 27^ and bicarbonate^28^. Extracellular factors that function as guidance cues to regulate growth cone development operate through the generation of localized intracellular raise of the second messengers cAMP and calcium^29^. Because CRH-activated CRHR1 has been shown to trigger an increase in calcium^9, 13^ which is critical for sAC activation, we investigated the involvement of calcium in the neuritogenic effect of CRH. In cells pre-incubated with the cell-permeable calcium chelator BAPTA-AM, the morphological change in response to CRH was significantly reduced (Fig. 3b). Simultaneous inhibition of calcium response and sAC activity impaired the neuritogenic effect of CRH to a similar extent, suggesting that calcium and sAC are involved in the same mechanism (Fig. 3b). This suggests that CRH-mediated neurite outgrowth depends on calcium, and it is consistent with the involvement of sAC in this process.

Next, we wondered whether a calcium rise was sufficient to trigger a cAMP response and neurite outgrowth. Treatment with thapsigargin, a blocker of sarcoendoplasmic reticulum calcium ATPase (SERCA) pumps, induced morphological changes in HT22-CRHR1 cells characterised by elongated neurites respect to basal (Supplementary Fig. 5a). Compared to CRH effect, the neuritogenetic effect of thapsigargin was less prominent (compare Fig. 2e and Supplementary Fig. 5a). We verified that thapsigargin raised calcium levels from intracellular stores (Supplementary Fig. 5b), but with a different temporal profile compared to the one evoked by CRH^9, 13^. Thapsigargin did not produce an increase in cAMP levels nor altered CRH-dependent cAMP response (Supplementary Fig. 5c). In addition, sAC-specific inhibitor KH7 had no effect on thapsigargin-dependent neurite outgrowth (Supplementary Fig. 5a). Calcium is a second messenger involved in the action of several neuritogenic stimuli^29^, which, as cAMP, is highly organized in signalling microdomains^30–32^. These results suggest that the coupling of CRH-evoked calcium to sAC (Fig. 3b) could not be mimicked by calcium originated by thapsigargin treatment, highlighting the importance of the cellular compartmentalization of signalling mediators for the cellular response^33^.

Finally, we assessed the effect of the sAC-specific activator bicarbonate on the neuritogenic effect of CRH. In previous experiments, the medium used was 25 mM bicarbonate, which reproduces the bicarbonate concentration *in vivo*. When HT22-CRHR1 cells were stimulated in bicarbonate-free medium, CRH-triggered neurite outgrowth was strongly reduced (Fig. 3c). Given that sAC is considered the only cellular target modulated by bicarbonate, these results further support a critical role for sAC in the neuritogenic effect of CRH.

### tmAC and sAC mediate CREB activation in response to CRH

The cAMP-response element binding protein (CREB), a key regulator of neuronal function, is the archetypal transcription factor targeted by cAMP. In response to CRH and UCN1, CREB was phosphorylated at S133 in a concentration dependent manner in HT22-CRHR1 cells (Supplementary Fig. 6). CREB activation increased over time of stimulation, achieving the maximal response about 30–40 min after CRH addition (Fig. 3d). In presence of the CRHR1 antagonist, DMP696, CREB phosphorylation was reduced confirming that it depends on CRHR1 activation (Fig. 3e).

We next asked whether both tmAC- and sAC-generated cAMP pools led to CREB activation. Using pharmacological inhibitors, we found that tmAC-specific 2′,5′-dideoxyadenosine (ddA), as well as sAC-specific KH7, reduced CRH-mediated CREB phosphorylation (Fig. 3f). Moreover, the simultaneous inhibition of tmACs and sAC led to a stronger reduction of phospho-CREB (Fig. 3f). Thus, CREB activation is dependent on the cAMP response triggered by CRHR1, being both tmAC and sAC involved in this process.

cAMP-mediated cell differentiation is characterised by the induction of specific genes through activated CREB. By q-RT-PCR we measured the expression of *c-fos* as an example of an endogenous CREB target gene. CRH produced a robust *c-fos* mRNA increase 1 h after stimulation, consistent with being an immediate early gene^34^. *c-fos* expression was significantly impaired when cells were pre-incubated with tmAC- or sAC-specific inhibitors (Fig. 3g). When both inhibitors were used in combination, a stronger reduction of *c-fos* expression was evidenced (Fig. 3g), reinforcing the model of distinct cAMP sources contributing to the overall CRHR1/cAMP dependent signalling cascade.

### CRH does not interfere with cell viability and induces cell cycle arrest

**Figure 4:**
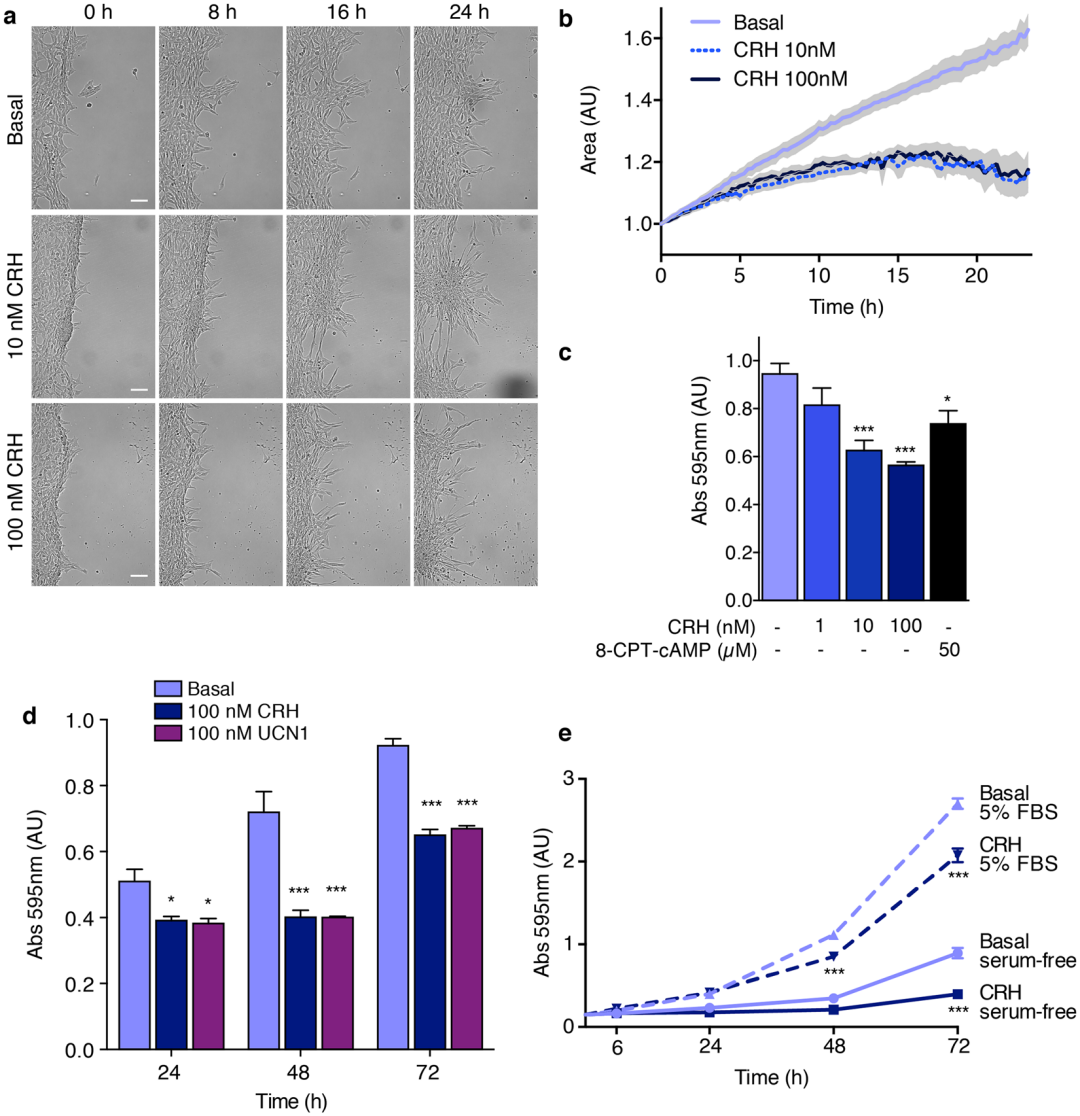
CRHR1 activation has a negative effect on cell proliferation. HT22-CRHR1 cells were stimulated with CRH, UCN1 or 8-CPT-cAMP at the indicated concentrations in presence of 1% FBS unless otherwise stated. (**a,b**) Effect of CRH on a “scratch” assay. (**a**) Representative photographs are shown for each treatment. Scale bars, 100 μm. (**b**) Quantification of the area occupied by cells along the treatment with respect to time 0. Data: mean ± SEM, n = 8. (**c–e**) Proliferation assay by crystal violet staining. Cells were stained with crystal violet at the indicated time points and the stained cells were dissolved for absorbance reading. Data: mean ± SEM, n = 3. *p < 0.05 ***p < 0.001 respect to basal in each condition by one-way ANOVA followed by Tukey post test.

There are several reports regarding CRH effect on cell proliferation but the observations are often contradictory, suggesting that this effect might be dependent on the experimental system used^35^. In cell models widely used to study neuritogenesis, such as rat pheochromocytoma PC12 cells and mouse neuroblastoma Neuro-2a cells, morphological changes are accompanied by cell growth arrest. In order to test whether CRH has a role in HT22-CRHR1 proliferation we used two experimental approaches. First, we performed an area-based growth test by a “scratch” assay. In this test, the gap area may be filled by a combination of cell motility and proliferation. When wound invasiveness is observed in presence of serum after long incubation times, as in the experiments shown (Fig. 4a,b), cell proliferation is considered to have the greatest impact.

HT22-CRHR1 monolayer was scratched to create a wound area free of cells and the cultures were imaged over time using bright field microscopy at the indicated times. In presence of CRH, the morphological change was evident in cells at the edge of the scratch (Fig. 4a). Notably, the total cell area covered by cells in the wound was significantly reduced compared to that of control conditions (Fig. 4b).

In addition, we tested cell survival and growth by a crystal violet assay. Crystal violet dye binds to proteins and DNA, thus, providing quantitative information about the density of attached cells. The effect of CRH on growth arrest was measured at different CRH concentrations, and was mimicked by the cell-permeable cAMP analogue, 8-CPT-cAMP (Fig. 4c). CRH and UCN1 stimulation induced HT22-CRHR1 growth arrest at a comparable rate (Fig. 4d).

We compared the effect of CRH to control conditions in cells cultured in a serum-free medium and in presence of 5% serum at different time points (6 h, 24 h, 48 h and 72 h) after CRH addition (Fig. 4e). While it was clear that serum stimulated cell proliferation, CRH promoted growth arrest with respect to the unstimulated control both in the presence and the absence of serum.

**Figure 5:**
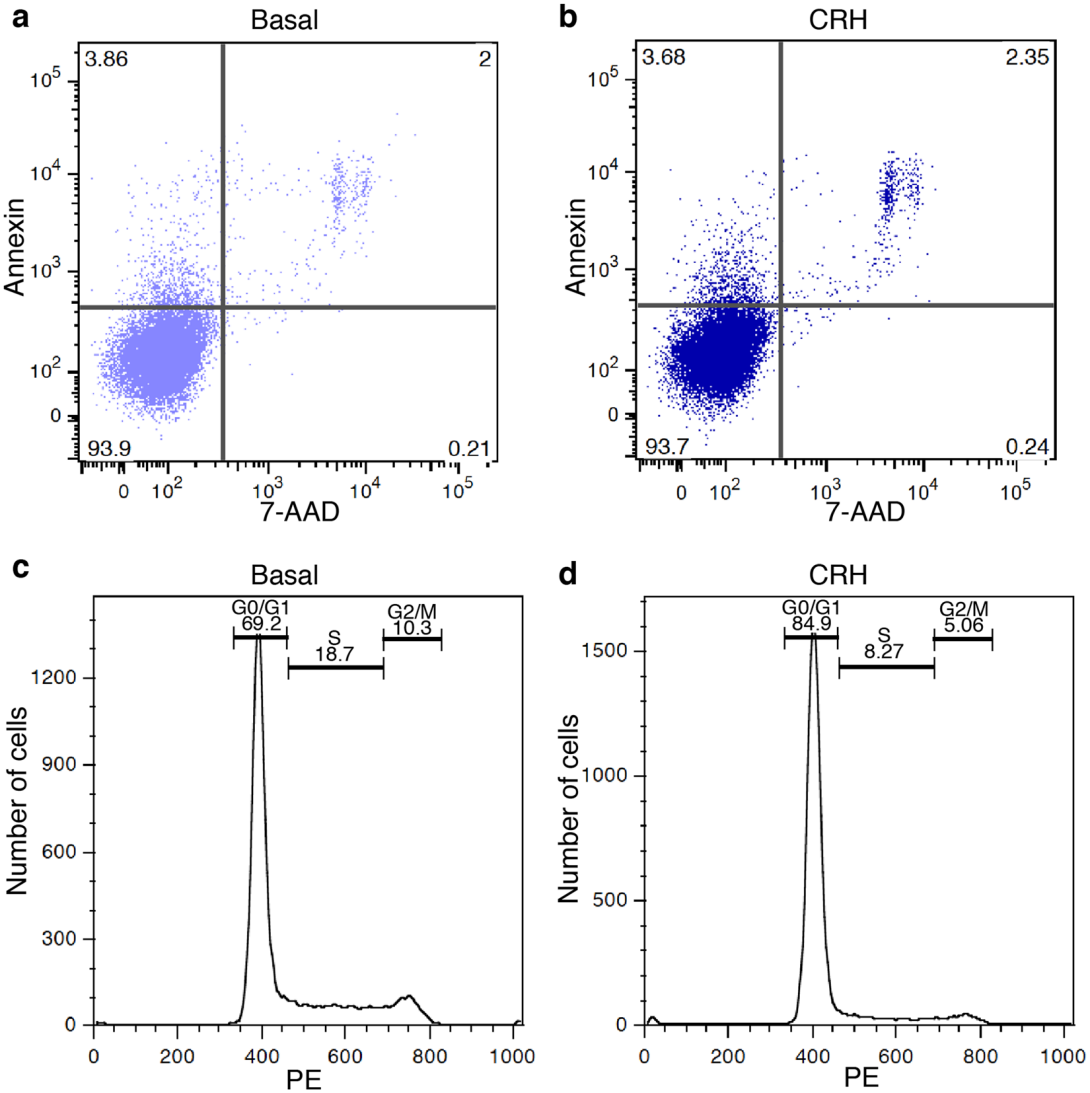
CRHR1 activation does not induce apoptosis but promotes cell cycle arrest. HT22-CRHR1 cells were mock (**a**,**c**) or CRH stimulated (**b**,**d**) for 24 hs. (**a**,**b**) Cells were double-stained with Annexin V/7-ADD and measured by flow cytometry to study apoptosis. Percentage of cells in each quadrant is shown. A representative dot plot from 3 independent experiments is shown. (**c,d**) Cells were stained with propidium iodide and analysed by flow cytometry to determine the cell-cycle progression. Representative flow cytometry histograms from 3 independent experiments are shown. Gates indicate the percentages of cells in various phases of the cell cycle (G_0_/G_1_, S, G_2_/M).

CRH-dependent effect on the number of cells may be achieved either through active promotion of apoptosis or through cell cycle arrest. We examined if CRH enhanced apoptosis measuring by flow cytometry Annexin-V binding and 7-AAD staining as early and late apoptotic markers, respectively. The proportion of viable and apoptotic cells was comparable between control and CRH-treated cells (Fig. 5a,b), suggesting that CRH is not a pro-apoptotic stimulus. In contrast, when we analysed CRH effect on cell cycle by propidium iodide staining, we observed that CRH incubation induced an accumulation of cells in G_0_/G_1_ phases (Fig. 5c,d). Taken together, these results suggest that CRHR1 activation leads to a signal transduction cascade that inhibits cell proliferation and activates differentiation.

### Serum antagonizes CRH-dependent HT22-CRHR1 differentiation

The regulation of cAMP levels by activated GPCR control multiple signalling pathways, which in turn, modulate diverse cellular functions. Also, it has long been appreciated that cAMP has a key role in cell growth and cell proliferation, being remarkable cAMP’s capacity to stimulate proliferation in some cell types, while it enhances cell differentiation in others^1^.

Considering that CRH promotes HT22-CRHR1 differentiation and that cAMP is critical in this process, we aimed to elucidate the downstream signalling pathways involved in CRH-elicited neuritogenesis. Complex intracellular signalling is involved in the neurite outgrowth process, and although many constituents are common in different cell types, notable differences have been identified among specific neuronal subtypes. Perhaps the best-characterised cell model regarding the cell-fate decision is the PC12 cell line, where transient ERK1/2 activation triggers proliferation whereas sustained ERK1/2 activation triggers differentiation^11^, and the ratio between activated ERK1/2 and AKT is critical in the all-or-none decision between proliferation and differentiation^36^.

**Figure 6:**
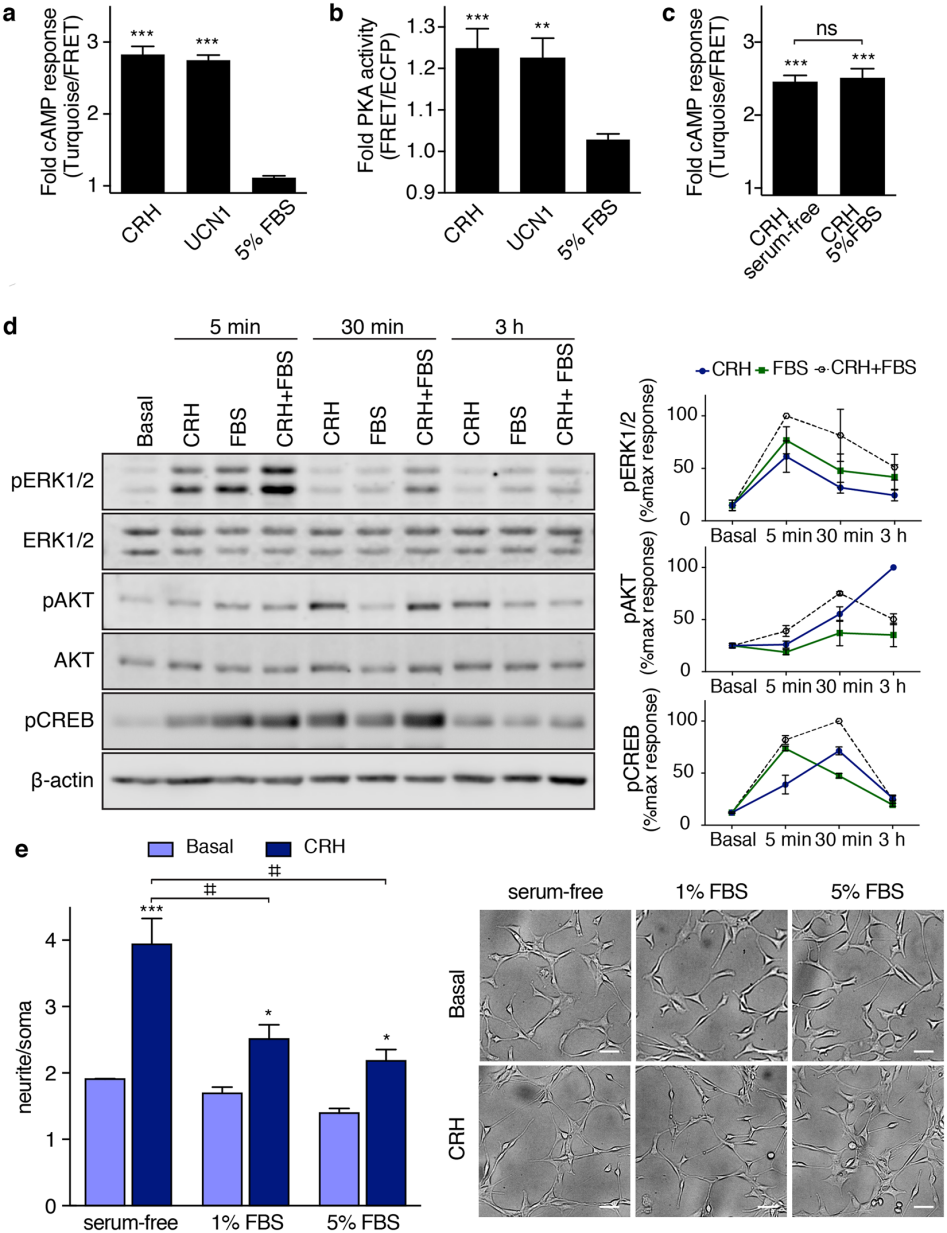
CRH- and serum-triggered responses in HT22-CRHR1 cells. (**a–c**) cAMP levels and PKA activity were determined as FRET changes in HT22-CRHR1 cells stably expressing Epac-S^H187^ or AKAR4 constructs, respectively. (**a**,**b**) Cells were stimulated with 100 nM CRH or UCN1, or 5% FBS in phenol red–free DMEM. (**c**) Cells were stimulated with 100 nM CRH in serum-free or 5% FBS phenol red–free DMEM. Bars represent the maximum FRET change respect to the basal (1 min after stimuli addition). Data: mean ± SEM, 20–25 cells from three independent experiments. *p < 0.05 ***p < 0.001 respect to basal in each condition by one-way ANOVA followed by Tukey post test. (**d**) HT22-CRHR1 cells stimulated with 100 nM CRH, 5% FBS or CRH and combination treatments at the indicated times points. Phosphorylated (pERK1/2) and total ERK1/2, phosphorylated (pAKT) and total AKT, phosphorylated CREB (pCREB) and actin were determined by Western blot. Results are expressed as the percentage of maximum response after stimulation. Data: mean ± SEM, n = 3. (**e**) Neurite outgrowth was quantified in HT22-CRHR1 cells stimulated with 100 nM CRH in serum-free media or in presence of 1% or 5% FBS. Data: mean ± SEM (n = 3). A representative photograph is shown for each treatment. Scale bars, 50 μm. Significant effects for CRH treatment (p = 0.0005) and for serum treatment (p = 0.0024) by repeated measures two-way ANOVA followed by Sidak post test (*p < 0.05 ***p < 0.001 respect to basal, ^‡‡^p < 0.01 between indicated treatments).

First, we explored if there was a crosstalk between the effect of CRH and the pathways activated by a proliferative stimulus, such as serum. Using the FRET-based biosensors Epac-S^H187^ (Fig. 6a) and AKAR4 (Fig. 6b), we determined that CRH and UCN1 triggered cAMP production and PKA activation to a similar extent, which is consistent with a similar effect on the morphological change (Fig. 2e). Conversely, the addition of serum did not affect cAMP levels or PKA activity in serum-starved HT22-CRHR1 cells (Fig. 6a,b). The cAMP response to CRH was similar in presence or absence of 5% FBS (Fig. 6c). We analysed the activation of ERK1/2, AKT and CREB by CRH, serum and both stimuli combined (Fig. 6d). CRH induced a strong phosphorylation of ERK1/2 at the early time point of 5 min and a small ERK1/2 response at 30 min and 3 h time points, consistent with the temporal profile of ERK1/2 activation in HT22-CRHR1 cells^13^. When serum was used as stimulus, ERK1/2 was also activated at the early time point (5 min) and modestly at 30 min and 3 h. It has been previously shown that a rise in cAMP leads to ERK1/2 activation in these cells^9^. Notably, the responses were additive when cells were stimulated with CRH and serum simultaneously, suggesting that CRH and serum activate ERK1/2 through different mechanisms.

CRH triggered a sustained AKT phosphorylation after 30 min, whereas serum had no detectable effect in this pathway at any of the time points analysed. It is to note that while the activation of the PI3K/AKT pathway promotes neurite outgrowth in a hippocampal context^37–39^, the stimulation of this pathway inhibits the differentiation of PC12 cells^36, 40^. CREB was phosphorylated by both CRH and serum to a similar extent at 5 and 30 min time points although the responses were stronger in cells simultaneously incubated with both stimuli, denoting different mechanisms involved in CREB activation by CRH and serum (Fig. 6d). Thus, it is possible to speculate about a cAMP-dependent and a cAMP-independent activation of CREB in response to CRH and serum respectively in HT22-CRHR1 cells.

Furthermore, CRH ability to induce HT22-CRHR1 neurite outgrowth was reduced in presence of increasing amounts of serum (Fig. 6e) by a cAMP-independent mechanism (Fig. 6c). Taken together, these results indicate that even though the signalling mechanisms triggered by CRH and serum are different, they are both capable of activating common molecular effectors such as ERK1/2 and CREB. However, serum and CRH exert opposite effects in HT22-CRHR1 cells neuritogenesis, suggesting that ERK1/2 activation is not sufficient to achieve the morphological change.

### CRHR1-mediated neurite outgrowth depends on PKA but not on ERK1/2 in HT22-CRHR1 cells

**Figure 7:**
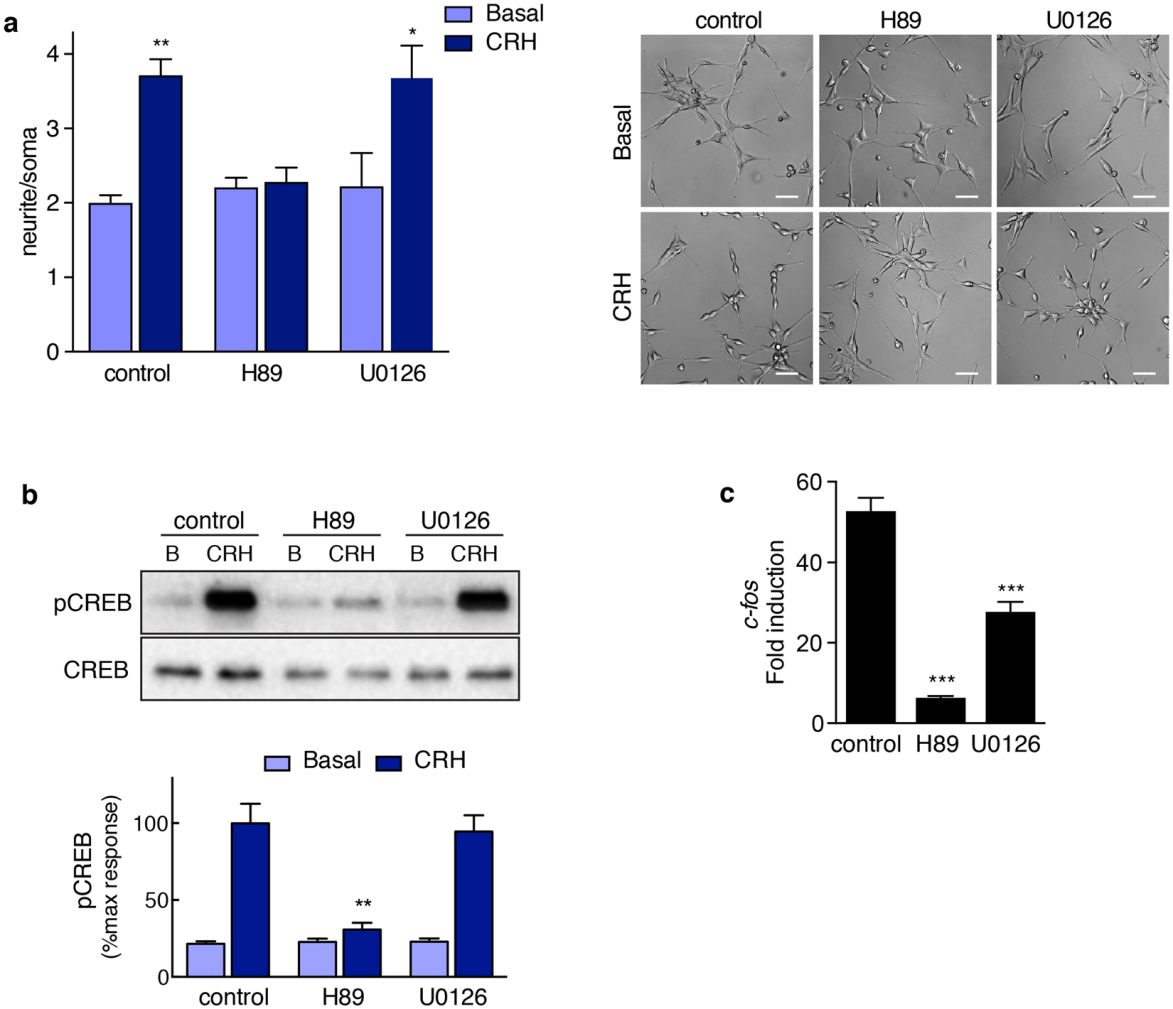
PKA activation is critical for CRH mediated cell differentiation and CREB phosphorylation. (**a**) Neurite outgrowth was determined in HT22-CRHR1 cells stimulated with 100 nM CRH in presence of vehicle (control), PKA-specific (10 µM H89), or MEK1/2-specific (10 µM U0126) inhibitors. Data: mean ± SEM (n = 3). ***p < 0.001 respect to basal, ^‡^p < 0.05 between indicated treatments by repeated measures one-way ANOVA followed by Tukey post test. A representative photograph is shown for each treatment. Scale bars, 50 μm. Cells were stimulated with 100 nM CRH in presence of vehicle (control), 10 µM H89, or 10 µM U0126. (**b**) phosphorylated CREB (pCREB) and total CREB were determined by Western blot in 40-min cell lysates. Results are expressed as the percentage of maximum pCREB after stimulation. Data: mean ± SEM, n = 3. (**c**) *c-fos* mRNA levels after 1 h were determined by RT-q-PCR and normalized to *Hprt*. Data: mean ± SEM, n = 3. ***p < 0.001 respect to control by one-way ANOVA followed by Tukey post test.

To study the signalling pathways involved in the CRH-mediated neurite outgrowth, we measured the morphological change when HT22-CRHR1 cells were pre-incubated with different pharmacological inhibitors. While PKA-specific inhibitor H89 abolished CRH-induced neuritogenic effect, no differences were found between control and MEK1/2 inhibitor U0126 pre-treated cells (Fig. 7a). CRH-dependent neurite outgrowth was also impaired in presence of a different PKA-specific inhibitor RpcAMPS, confirming the role of PKA in this process (Supplementary Fig. 7).

Using the PC12 cell line, it has been extensively studied that the sustained activation of ERK1/2 in response to growth factors is essential to trigger differentiation. A characteristic of neuronal and endocrine cellular contexts is that GPCR-dependent ERK1/2 activation takes place downstream the cAMP response, as we have shown it is the case for HT22-CRHR1 cells^9^. On the other hand, platelet-derived growth factor (PDGF), which signals via a RTK, also activates ERK1/2 in HT22-CRHR1 cells^13^. We observed that PDGF induced neurite outgrowth in HT22-CRHR1 cells (Supplementary Fig. 8a). However, whereas CRH neuritogenic effect was independent of ERK1/2 activation, PDGF neuritogenic effect was blocked in presence of the MEK1/2 inhibitor U0126 (Supplementary Fig. 8a). As we described for CRH-dependent neurite outgrowth (Fig. 6e), a proliferative stimulus such as FBS also antagonized the PDGF-dependent neuritogenic effect (Supplementary Fig. 8b), even though PDGF and serum are both capable of activating ERK1/2 in this cell line.

It is to note that phospho-ERK1/2 in response to CRH or PDGF display different subcellular localizations suggesting that different ERK1/2 activated pools are generated from each stimulus^13^. Remarkably, PDGF did not raise cAMP levels in HT22-CRHR1 cells (Supplementary Fig. 8c), which is consistent with a cAMP-independent ERK1/2 activation by growth factors. Thus, different neuritogenic stimuli as CRH and PDGF can activate common effectors (for example, ERK1/2) with different roles regarding cell differentiation. Collectively, these data show that ERK1/2 is capable to mediate morphological changes in HT22-CRHR1 cells, but the phospho-ERK1/2 downstream of CRHR1 activation is not involved in this effect.

### PKA but not ERK1/2 regulates CREB activation in response to CRH

We next sought to determine the involvement of PKA and ERK1/2 in CRH-dependent CREB phosphorylation. When cells were pre-treated with PKA inhibitor H89, CREB phosphorylation was blocked confirming that PKA regulates cAMP-dependent CREB activation, but phospho-CREB was not affected when cells were pre-treated with U0126 (Fig. 7b). In presence of two different MEK1/2 inhibitors, U0126 and PD98059, CRHR1-mediated ERK1/2 activation was completely abolished (Supplementary Fig. 9a) while no differences were observed in CREB activation when cells were stimulated with CRH or UCN1 (Supplementary Fig. 9b). This is in line with previous studies showing that ERK1/2 activation is not required for CRH-mediated CREB phosphorylation in hippocampal neurons^41^.

Finally, we assessed PKA and ERK1/2 effect in *c-fos* expression in response to CRH. Whereas PKA inhibition prevented CRH-mediated *c-fos* induction, we observed that *c-fos* expression was also diminished in presence of the MEK1/2 inhibitor (Fig. 7c). Therefore, although ERK1/2 is not involved in CREB phosphorylation, ERK1/2 seem to be at least in part required for CRHR1/cAMP transcriptional effects.

## Discussion

The key role of cAMP in the regulation of cell differentiation has been the subject of intense investigation. In neuronal models, cAMP capacity to enhance the outgrowth of neuronal processes has received special attention. Our present findings show that CRHR1 activation promotes growth arrest and the elongation of neurites in HT22-CRHR1 cells. We analysed the neuritogenic effect to identify the molecular mechanisms involved, in order to get further insight into pathways activated downstream of CRHR1. We demonstrate that the cAMP/PKA signalling pathway is critical for CRH-dependent neurite outgrowth, but ERK1/2 phosphorylation is dispensable for this process. The cAMP/PKA response to CRH stimulation in HT22-CRHR1 depends not only on tmACs but also on sAC activity^9^. Our present results further highlight the role of two sources of cAMP downstream the activation of a GPCR, showing that tmAC as well as sAC are involved in CRH-mediated CREB phosphorylation and *c-fos* induction. Remarkably, only sAC-generated cAMP pools proved critical for the neuritogenic effect of CRH, reinforcing the notion that restricted cAMP microdomains may regulate independent cellular processes.

We have recently reported that sAC represents an alternative source of cAMP downstream a GPCR in addition to classical tmAC, focusing on the role of different cAMP sources in ERK1/2 activation mechanisms in response to CRH in HT22-CRHR1 cells^9^. Furthermore, we have demonstrated that sAC-generated cAMP is specifically involved in cAMP generation after CRHR1 internalization and required for the sustained “endocytic” phase of ERK1/2 signalling^9^. Here, we provide additional evidence of a functional diversification between tmACs and sAC. Collectively, our previous and present results show that the activity of both tmACs and sAC is necessary for classical components of cAMP signalling such as PKA activation, early ERK1/2 activation^9^, CREB phosphorylation and *c-fos* transcription. In contrast, we report that sAC-generated cAMP is the one responsible for CRH-mediated morphological change in HT22-CRHR1 cells and that the acute activation of sAC, which regulates PKA, is essential for the neuritogenic effect of CRH.

**Figure 8:**
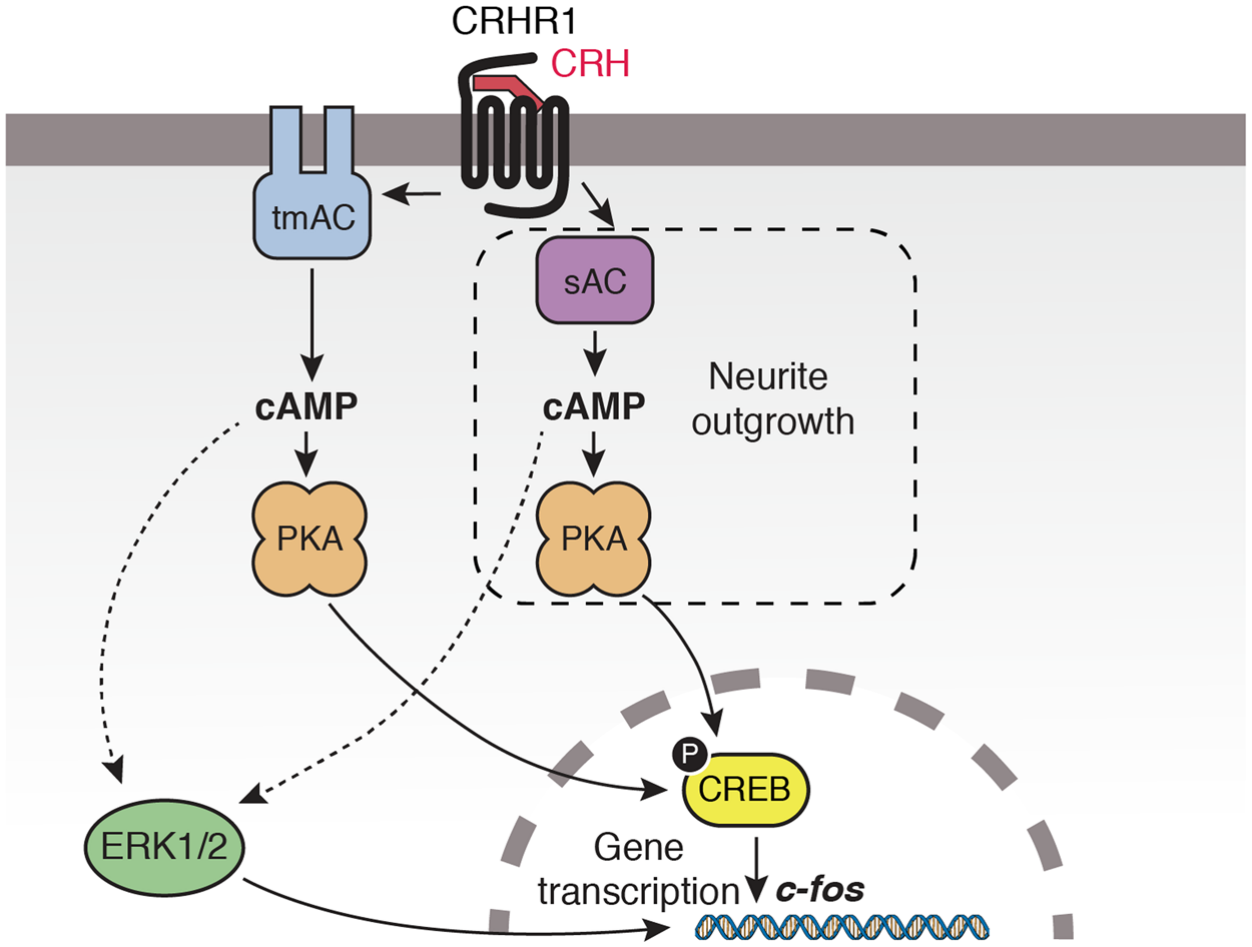
Proposed model for CRHR1 signalling involved in cell differentiation. In HT22-CRHR1 cells, activated CRHR1 generates cAMP through tmACs and sAC, which engages PKA and leads to ERK1/2 and CREB activation. sAC activity generates the essential cAMP pool required for ERK1/2-independent neurite outgrowth. Both phospho-CREB and activated ERK1/2 are required for CRH-regulated gene transcription of the early gene *c-fos*.

The observation that ERK1/2 activation is dispensable for CREB phosphorylation and neurite outgrowth in response to CRH also highlights the existence of a complex network of biochemical routes (Fig. 8). For example, tmACs and sAC mediate PKA activation, which is involved in the phosphorylation of both ERK1/2 and CREB, although these pathways are functionally insulated signalling paths. In addition, only sAC-activated PKA pool seems to be involved in the neuritogenic effect of CRH. Regarding ERK1/2 role in neuritogenesis, in this work we show that ERK1/2 has the capacity to regulate morphological changes in these cells, as in response to PDGF, but phospho-ERK1/2 is not essential for CRH-mediated neurite outgrowth. Moreover, a proliferative stimulus, such as serum, also induced a similar ERK1/2 activation and had an opposite role to CRH with respect to morphological changes and cell proliferation. Prolonged ERK1/2 activation is sufficient for PC12 cell differentiation, but the results obtained in PC12 cells are not generally translated to hippocampal cells. It has been shown that ERK1/2 activation is not required for differentiation nor CREB phosphorylation in immortalized hippocampal cells^42, 43^ and in primary hippocampal cells, CRH triggered CREB phosphorylation independently from ERK1/2^44^. Even in PC12 cells, CREB activation in response to GPCR ligand PACAP is independent from ERK1/2^45^. Thus, in this hippocampal cell model HT22-CRHR1, we can identify multiple cAMP-dependent pathways for activated CRHR1 in the same cell, some of them cross-regulated and others insulated from one another: tmAC/sAC-PKA-dependent (early phospho-ERK1/2); sAC-dependent, PKA-independent (late phospho-ERK1/2); tmAC/sAC-PKA-dependent, ERK1/2-independent (CREB activation); sAC-PKA-dependent, ERK1/2-independent (neurite outgrowth).

Which is the physiological role of activated CRHR1 on neuronal proliferation and differentiation? Previous reports exploring the role of CRH in the regulation of cell proliferation suggest that the effect is not general but specific with respect to the cellular context: an anti-proliferative CRH effect was reported in neuroblastoma SK-N-SH cells^46^ and tumour cell lines derived from pituitary, endometrium and breast^47–50^ whereas a CRH-favoured tumorigenic action was described in epidermis and gastric cancer models^51, 52^. CRH enhanced proliferation of neuronal progenitors^53^, and regulated growth of different skin cell types^35^. The evidence available regarding CRH as a modulator of neuronal architecture also remains controversial. CRHactivated CRHR1 promoted neurite outgrowth via a PKA- and ERK1/2-dependent mechanism in the noradrenergic locus coeruleus-like CATH.a cell line, locus coeruleus organotypic slices, and Purkinje cells in cerebellar slices^54–56^ but reduced dendritic arborisation of neurons of the developing hippocampus^57^. CRH increased the spines in the cerebellum slices^58^ whereas CRH disrupted the thin spines in hippocampal slices^59^. In some systems, CRH and UCNs exerted similar effects^55, 58^ but also opposing roles have been reported in others^56^. The difference between previous findings and ours might result from the differences in the experimental system such as the type of cells used or culture and stimulus conditions. In this work we focused on the morphological change in HT22-CRHR1 cells as a bioassay to elucidate signalling mechanisms involved in CRHR1 function in neurons. Studies in established cell lines, such as those we report here and previous works from other groups described here, are far from being considered of direct physiological significance. However, HT22-CRHR1 cells maintain essential features of the cAMP response to CRH (Fig. 1) and proved to be an *in vitro* model useful to perform molecular and cellular experiments that would be more complex, difficult, and even unfeasible, *in vivo* ^9, 13, 60^. In addition, the neuritogenic and anti-proliferative effect observed for CRH and UCN1 by CRHR1 activation in this cellular system provide valuable information on a subject that is still an open question.

Because the establishment of neuronal connectivity is crucial for brain function, the action of CRH within the CNS as a modulator of synaptic plasticity and neuronal networks during development and stress-related disorders might account for long-lasting effects of stress responses^61^. Future studies assessing the role of RhoGTPases regulation in response to CRH need to be carried out to define the cytoskeleton dynamics in CRHR1 triggered neuronal morphological changes^29^.

The importance of sAC mediating neurite outgrowth and neuronal survival has become recently appreciated, although the upstream components that activate sAC and the sAC-dependent mechanisms engaged remain to be fully defined (reviewed in ref. 62). In cultured dorsal root ganglion cells (RGCs), sAC was involved in the axonal outgrowth and growth cone elaboration in response to netrin-1, a critical guidance cue for neurons^21^. No aberrant axon guidance was observed during development in the sAC C1 knockout mice^63^, suggesting the existence of complementary or redundant mechanisms^64^. In addition, sAC was shown to promote RGC survival and axon growth in response to electrical activity whereas other calcium-responsive tmACs (AC1 and AC8) had no effect in these functions^23^. A role of sAC has been also suggested for the signalling of neurotrophins, which activate RTK triggering multiple intracellular signalling pathways through protein-protein interactions and regulate structural changes in neurons^65^. Increasing evidence shows that cAMP regulates RTK-mediated guidance cues^66–68^ but because RTKs do not activate tmACs directly, the mechanism that leads to cAMP has remained elusive. It was reported that sAC mediates NGF-dependent Rap1 activation^69^, and mediates morphological changes in PC12 cells^70^. Additionally, it was shown that BDNF-induced axonal outgrowth on MAG/myelin depends on sAC activity^71^.

To our knowledge this is the first description of sAC-generated cAMP promoting morphological changes downstream of a GPCR. Our report that sAC participates in CRHR1 activated processes relevant for neuronal function, like neuritogenesis, CREB phosphorylation and *c-fos* induction, provides evidence that sAC is not only involved in “atypical” cAMP mechanisms (RTKs and netrin responses, for example), but also in canonical cAMP pathways, such as those elicited by GPCRs. Given that sAC is directly activated by calcium, it is of special interest to investigate its role in potential mechanisms that integrate networks of both second messengers, cAMP and calcium, which govern most of neuronal cellular functions^5, 29^. In this regard, it is important to note that cAMP and tmACs role in neuritogenesis and neuronal survival have been classically studied using forskolin. Although sAC is insensitive to forskolin, the whole-cell cAMP increase in response to this reagent does not account for the activation of spatially regulated cAMP microdomains observed under physiological stimuli. Further studies to characterise the individual roles of different ACs will be valuable to understand the compartmentalization and diversification of the signals inside the cell.

## Materials and Methods

### Cell culture and transfection

HT22 stable clones expressing cMyc-CRHR1 were previously described^13^. Parental HT22 cells, HT22-CRHR1 cell line, HT22-CRHR1 clones stably expressing Epac-S^H^ ^187^ or AKAR4^9^ were cultured in DMEM supplemented with 5% fetal bovine serum (FBS), 2 mM L-glutamine, 100 U/ml penicillin and 100 μg/ml streptomycin (Invitrogen) at 37 °C in a humidified atmosphere containing 5% CO_2_.

Plasmids were transfected using Lipofectamine and Plus Reagent according to the manufacturer’s instructions and as previously described^13^. Experiments were performed 48 h after plasmid transfection. mTurquoise2-EPAC-cp173Venus-Venus (Epac-S^H187^) construct was kindly provided Dr. K. Jalink (Department of Cell Biology, The Netherlands Cancer Institute, The Netherlands); AKAR4 by Dr. J. Zhang (Department of Pharmacology and Molecular Sciences, Johns Hopkins University, USA).

### Animals

Mice were housed under standard laboratory conditions (22 ± 1 °C, 55% ± 5% humidity) with food and water ad libitum. Animal experiments were conducted in accordance with the Guide for the Care and Use of Laboratory Animals of the Government of Upper Bavaria (Germany) and approved by the Animal Care and Use Committee of the Max Planck Institute of Psychiatry (Munich, Germany).

### Primary cultures and transfection

Wild-type (WT) primary hippocampal and cortical neurons were prepared from CD1 mouse embryos (E17.5–18.5). Primary cell cultures lacking CRHR1 in glutamatergic neurons were prepared from embryos derived from breeding *Crhr1* ^*loxP/loxP*^; *Nex-Cre* (*CRHR1* ^*CKO-Glu*^) mice to *Crhr1* ^*loxP/loxP*^; *R26* ^*CAG::LSLtdTomato/CAG::LSLtdTomato*^ (*CRHR1* ^*CKO-Ctrl*^; Ai9) mice^7, 17^. Pooling of primary neurons from *Crhr1* ^*loxP/loxP*^; *R26* ^+*/CAG::LSLtdTomato*^; *Nex-Cre* and *Crhr1* ^*loxP/loxP*^; *R26* ^+*/CAG::LSLtdTomato*^ embryos resulted into 50% of glutamatergic neurons labelled by tdTomato and simultaneously lacking CRHR1. Primary cultures were maintained in Neurobasal-A medium with 2% B27 and 0.5 mM GlutaMAX-I (Gibco) at 37 °C and 5% CO_2_. Neurons were plated on coverslips (Menzel) coated with 50 μg/ml poly-D-lysin (Sigma) and 5 μg/ml laminin (Invitrogen) at a density of 65,000 cells per coverslip. Neurons were transfected via a calcium phosphate protocol^72^.

### Ligand stimulation, drugs, and pharmacological inhibitors

Serum-starved cells were stimulated with human/rat CRH (H-2435, Bachem), forskolin (F6886, Sigma), 8-CPT-cAMP (F1221, Sigma), PDGF (01–305; Millipore) or fetal bovine serum (FBS, Natocor) at the concentrations and time points indicated. After incubations, cells were washed with ice-cold PBS and maintained in ice. When calcium chelator BAPTA-AM (B6769, Life Technologies), antagonists or pharmacological inhibitors were used, cells were pre-treated with the drugs or vehicle 15–30 min before stimulation. CRHR1-specific antagonist DMP696 was a generous gift from Dr. Hausch (Max Planck Institute of Psychiatry, Munich, Germany). The following inhibitors were used: H89 (PKA; 371963 Calbiochem), RpcAMPS (PKA; 1337, Tocris), 2′,5′-dideoxyadenosine (tmACs; 288104, Calbiochem), KH7 (sAC; 3834, Tocris), 2-hydroxyestradiol (sAC; 13019, Cayman), U0126 (MEK1/2; 662005, Calbiochem), PD98059 (MEK1/2; 1213, Tocris). For Western blot assays cells were serum-starved for 6 h in OptiMEM before drug pre-treatments or stimulation.

### Preparation of cellular extracts and immunoblotting

After treatments, cells were washed with ice-cold PBS and lysed in Laemmli sample buffer. Whole-cell lysates were sonicated and heated to 95 °C for 5 min. Samples were resolved by SDS-PAGE and transferred onto 0.45 mm nitrocellulose membranes (Millipore) for immunoblotting. Membranes were blocked in TBS-Tween 20 (0.05%) containing 5% milk at room temperature for 1 h under shaking and probed overnight at 4 °C with the primary antibodies. The following antibodies were used: anti–phospho-ERK1/2 (E-4, sc-7383) from Santa Cruz Biotechnology; anti–total-ERK1/2, (9102, Cell Signaling), anti-phospho CREB (06-519, EMD Millipore), anti-total-CREB (9104, Cell Signaling), anti-phospho AKT (4058, Cell Signaling), anti-total-AKT (2920, Cell Signaling).

Signals were detected by HRP-conjugated secondary antibodies and enhanced chemiluminescence (SuperSignal West Dura, Pierce) using a GBOX Chemi XT4 (Syngene) or by IRDye700DX and IRDye800CW secondary antibodies (Rockland). Phosphorylation of MAPK and CREB was detected with the Odyssey Fc Imaging System (Li-Cor Biosystems). Phosphorylated proteins were relativized to its total protein level and results expressed as the percentage of maximum pERK1/2 after stimulation. Immunoreactive signals were analysed digitally using Fiji software.

### Neurite outgrowth assay

Cells seeded in a 40% density in 12-well plates were stimulated with 100 nM CRH or UCN1, 50 µM 8-CPT-cAMP, 50 µM forskolin, 500 µM IBMX or 10 µM isoproterenol in the presence of vehicle or specific inhibitors in OptiMEM. After 20 h-treatment, cells were imaged under bright field illumination using an Olympus IX81 inverted epi-fluorescence microscope using a 20X air objective and Metamorph software for image acquisition. For each treatment, at least 15 random fields were imaged. Morphological changes quantification was performed using Simple Neurite Tracer plugin for FIJI software. Neurite outgrowth was determined as the ratio between the longest neurite and the soma diameter per cell after 20 h, measuring at least 100 cells per treatment. For statistical analysis, repeated measures one- or two-way ANOVA followed by the indicated post test (n = 3) were performed.

### Wound healing assay

Cells were cultured in 24-well plates to confluence. Wounds were created with a pipette tip and washed to remove cell debris. Cells were stimulated with 10 nM or 100 nM CRH in DMEM 1% FBS. Images were acquired with a Zeiss Axio Observer Z1 Inverted Epi-fluorescence microscope, equipped with an AxioCam HRm3digital CCD camera; a Stage Controller XY STEP SMC 2009 scanning stage and an Incubator XLmulti S1 and Heating Unit XL S1 for live imaging incubation.

Images were acquired under bright field illumination every 15 min for 24 h using a 10X air objective and Zeiss Zen Blue 2011 software for image acquisition. Image analysis was performed with Fiji software, using an automated analysis macro to measure the area occupied by cells.

### Crystal violet proliferation assay

Cells seeded in a 25% density in 24-well plates were stimulated with 100 nM CRH or UCN1, 50 µM 8-CPT-cAMP or vehicle for the indicated times. Then, medium was removed, cells were rinsed with ice-cold PBS and fixed with methanol for 15 min. Staining was performed with 0.5% crystal violet in water for 15 min. Plates were rinsed with water, dried out and the remaining crystal violet was solubilized in 200 µl methanol. The absorbance was measured at 595 nm in a plate reader.

### Flow cytometry-based apoptosis and cell cycle detection

Cells seeded in 6-well plates were stimulated for 24 h with 100 nM CRH or vehicle in OptiMEM. Cells were rinsed with PBS, trypsinized and collected by centrifugation.

Apoptosis was assessed by phosphatidylserine exposure analysis using PE-Annexin V and 7-AAD staining (BD Biosciences) according to manufacturer’s instructions. After 30-min incubation, samples were analysed by flow cytometry (BD Biosciences) to determine the proportion of apoptotic cells.

For cell cycle analysis, cells were washed with PBS and fixed with 70% ethanol added dropwise. Then, cells were washed with PBS and stained with propidium iodide (PI) solution containing 50 µg/ml PI and 50 µg/ml ribonuclease A for 30 min at room temperature. Stained DNA was analysed by a flow cytometer.

Flow cytometry data were acquired on a FACsCANTO II (BD Biosciences). Data were analysed using FlowJo software (Tree Star).

### RT-PCR and quantitative real-time PCR

Total RNA was extracted from cell lines, primary cultures or brain extracts using TRIzol reagent (Invitrogen) and complementary DNA synthesis was carried out using M-MLV reverse transcriptase in the presence of RNasin RNase inhibitor (Promega). PCR primers are all intron spanning. Quantitative real-time PCR was performed with Taq DNA polymerase (Invitrogen) and SYBR Green I (Roche) using a CFX96 Touch™ Real-Time PCR Detection System. Relative expression was calculated for each gene by the Ct method with *Hprt* for normalization. Sequences and expected product sizes are as follows: *Crhr1* sense 5′-GGGCCATTGGGAAACTTTA-3′, antisense 5′-ATCAGCAGGACCAGGATCA-3′ (109 bp); *sAC* sense 5′-CCTGCATCGCTGTCTGGTAT-3′, antisense 5′-GAACTGTCGGGGTTCTTCGT-3′ (102 bp); *c-fos* sense 5′-ATCGGCAGAAGGGGCAAAGTAG-3′, antisense 5′-GCAACGCAGACTTCTCATCTTCAAG-3′ (172 bp); *Hprt* sense 5′-TGGGCTTACCTCACTGCTTTCC-3′, antisense 5′-CCTGGTTCATCATCGCTAATCACG-3′ (139 bp).

### Spectral Förster Resonance Energy Transfer (FRET) live imaging of the cAMP response

HT22-CRHR1 cells expressing FRET biosensors were seeded in glass-bottom dishes. Cell imaging was performed on an inverted Zeiss LSM 710 confocal microscope (Carl Zeiss Microscopy GmbH) and ZEN Black 2011 software as previously described^9^. Images were acquired with a 40x/1.2 water immersion and temperature corrected objective lens at 1024 × 1024, 16 bit, pixel dwell time of 3.15 μs, with open pinhole (600 μm). For FRET experiments, cells were illuminated with a 30 mW 405 nm diode laser at 2% laser power, a 405 nm dichroic mirror was used and the emission was collected between 413–723 nm wavelength, every 15 s for a duration of 15 min. The saturation level was verified for each image.

Primary hippocampal and cortical neurons transfected with Epac-S^H187^ were grown on coverslips and transferred to an Attofluor chamber (Invitrogen). Neurons were imaged with an inverted Olympus IX81 confocal microscope and Fluoview 1000 software. Images were acquired with a 20X objective at 1024 × 1024, 12 bit, pixel dwell time of 4 μs, with open pinhole (800 μm). For FRET experiments, cells were illuminated with a 30 mW 405 nm diode laser at 5% laser power, and the emission was collected between 460–500 nm (Turquoise) and 515–615 (Venus) wavelengths, every 15 s for a duration of 15 min. The saturation level was verified for each image and probe saturation was evaluated stimulating with forskolin after CRH (Supplemental Fig. 1).

Phenol red–free DMEM/F12 medium supplemented with 20 mM HEPES was used and imaging was performed at 37 °C and 5% CO_2_. Around 2.5 min after the start of the experiment, CRH, FBS or UCN1 were added to the final concentration indicated. The cAMP response is shown as time courses or as bars, in which the maximum response measured in a 20-min interval is presented. The data is expressed as the fold response with respect to basal levels or as percentage of the maximum response, being 100% CRH-elicited cAMP in control conditions.

### Calcium imaging

Cells plated in glass-bottom dishes were loaded for 30 min in darkness with 6 µM Fluo-4-AM and 0.14% Pluronic F-127 (Molecular Probes) in Ringer buffer. Images were acquired with a Axio Observer Z1 inverted epi-fluorescence microscope (ZEISS), equipped with an AxioCam HRm3 digital CCD camera, a Stage Controller XY STEP SMC 2009 scanning stage, and an Incubator XLmulti S1 (D) and Heating Unit XL S1 (D) for live-imaging incubation. Data acquisition was controlled by Zen Blue 2011 software (ZEISS), configured at a bit depth of 14 bits. Cells were imaged with a 20X air objective (Plan-Apochromat NA 0.8 M27) and illuminated using Colibri.2 470-nm LED excitation (5% power), with a 50-ms exposure acquired every 5 s and a 38HE Filter. Image analysis was performed with Fiji by measuring calcium-dependent changes in fluorescence intensity from resting levels (ΔF/F0) in 30–40 cells randomly selected in each experiment.

### Statistics

Each experiment was performed at least 3 independent times. The results are presented as the mean ± SEM of each measurement. Comparisons between treatments were performed using Student’s t-test, one- or two-way ANOVA (GraphPad Prism) followed by *post-hoc* tests stated in the Figures. Statistically significant differences are indicated.

## Electronic supplementary material

Supplementary Information

Supplementary Video 1

Supplementary Video 2

Supplementary Video 3
