# Supplementary material for: cAMP-dependent cell differentiation triggered by activated CRHR1 in hippocampal neuronal cells
Source: Sci Rep. 2017 May 16;7:1944. doi: 10.1038/s41598-017-02021-7 (PMC5434020; doi:10.1038/s41598-017-02021-7)
Supplement: Supplementary file 1 — Supplementary Information [file 41598_2017_2021_MOESM1_ESM.pdf]

Supplementary Information

cAMP-dependent cell differentiation triggered by activated CRHR1 in hippocampal neuronal cells

Authors

Carolina Inda[1,2], Juan José Bonfiglio[1,4], Paula A. dos Santos Claro[1,2], Sergio A. Senin[1], Natalia G. Armando[1], Jan M. Deussing[3] & Susana Silberstein[1,2*].

Institutions

[1] Instituto de Investigación en Biomedicina de Buenos Aires (IBioBA)-CONICET-Partner Institute of the Max Planck Society, Buenos Aires, Argentina.

[2] DFBMC, Facultad de Ciencias Exactas y Naturales, Universidad de Buenos Aires, Buenos Aires, Argentina.

[3] Max Planck Institute of Psychiatry, Department of Stress Neurobiology and Neurogenetics, Molecular Neurogenetics, Munich, Germany

[4] Present address: Max Planck Institute for Biology of Ageing, Cologne, Germany

[*]Corresponding author

Susana Silberstein (ssilberstein@ibioba-mpsp-conicet.gov.ar): Instituto de Investigación en Biomedicina de Buenos Aires (IBioBA)-CONICET-Partner Institute of the Max Planck Society, Godoy Cruz 2390, C1425FQA Buenos Aires, Argentina; Tel: 54-11-4899-5500.

Supplementary Videos

Differential interference contrast (DIC) images were acquired using a Zeiss Axio Observer Z1 Inverted epi-fluorescence microscope equipped with a heat and CO_2 incubation chamber for live imaging. Images were acquired under bright field illumination every 20 min for 24 h using a 20X air objective and Zeiss Zen Blue 2011 software for image acquisition.

Supplementary Video 1 I HT22-CRHR1 cells incubated under basal conditions.

Supplementary Video 2 I HT22-CRHR1 cells stimulated with 100 nM CRH at time 0.

Supplementary Video 3 I HT22-CRHR1 cells stimulated with 100 nM UCN1 at time 0.

Supplementary Figures

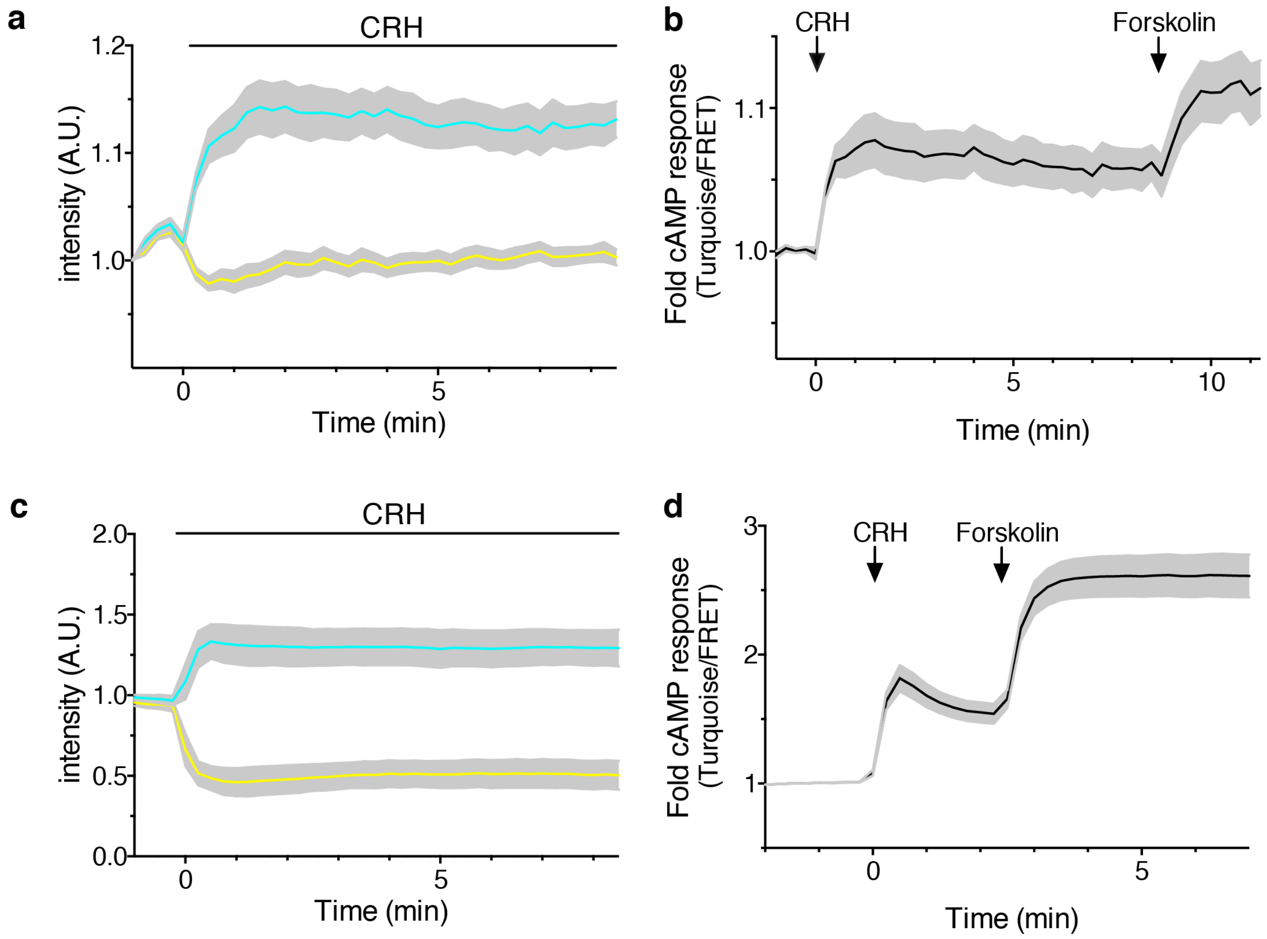

Supplementary Figure 1 I Optimization of cAMP measurements in HT22-CRHR1 and primary cultures

a-b, Time courses of the donor (mTurquoise2) and FRET mean intensity changes were determined relative to basal in single cells transfected with Epac-S^{H187} construct. **a,** Responses measured for primary hippocampal neurons corresponding to Figure 1c. **b,** Responses measured for HT22-CRHR1 cells corresponding to Figure 1e. Traces are representative of three independent experiments (mean ± SEM, 20–25 cells). A.U., arbitrary units.

c-d, Time course of FRET changes were measured in single cells transfected with Epac-S^{H187} construct. Primary hippocampal neurons (**c**) and HT22-CRHR1 cells (**d**) were analysed. Cells were stimulated with CRH (**c**, 100 nM or **d**, 10 nM) subsequently with forskolin (50 µM) at the indicated times. Traces are representative of three independent experiments (mean ± SEM, 20–25 cells).

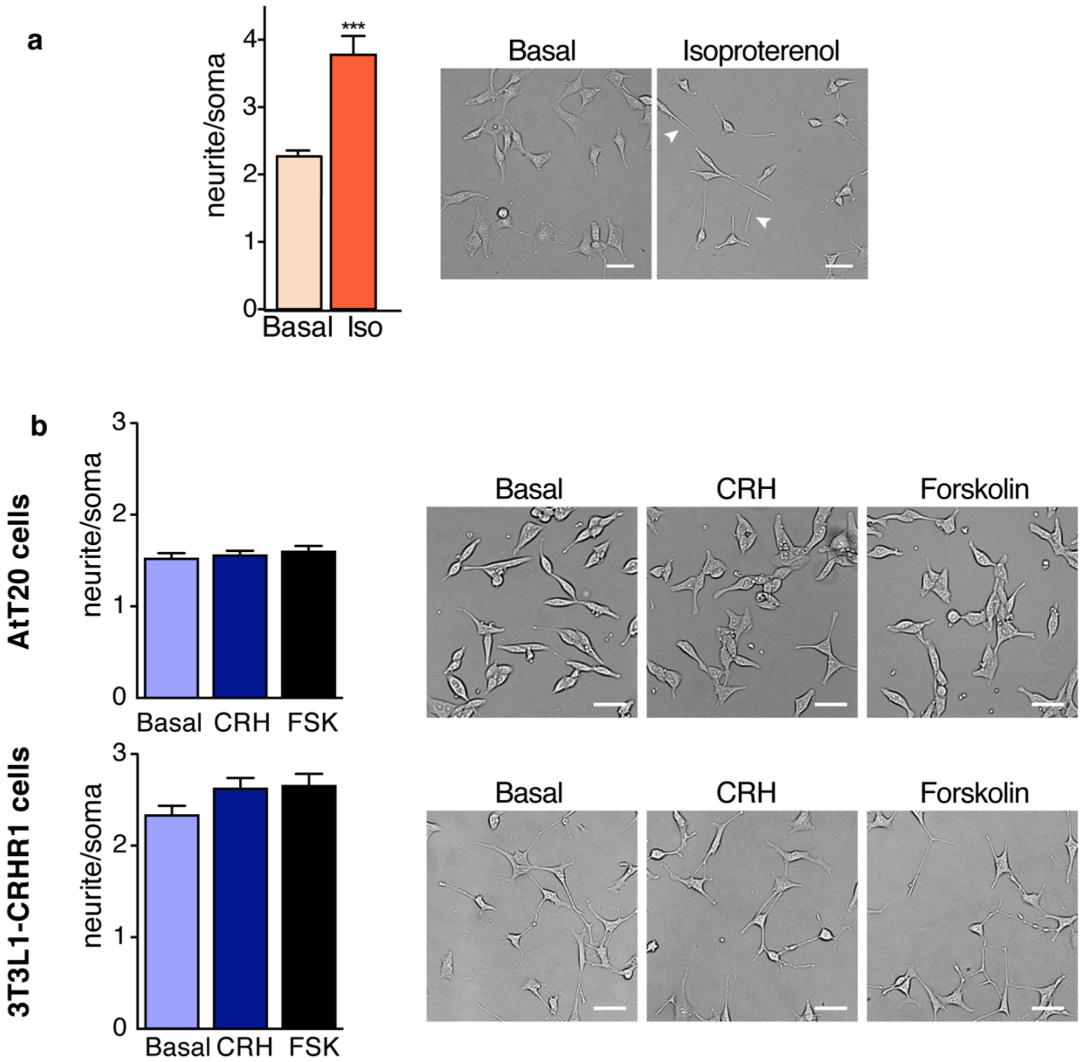

Supplementary Figure 2 I Neurite outgrowth effect of isoproterenol in HT22-CRHR1 cells and of CRH in other cell lines, AtT20 and 3T3L1-CRHR1 cells

a, Neurite outgrowth was quantified in HT22-CRHR1 cells stimulated with 10 µM isoproterenol (Iso). Data: mean ± SEM, n = 4. ***, $p < 0.001$ by Student's t test. Arrowheads point to neurite extensions.

b, Neurite outgrowth was quantified in AtT20 cells (top panel) and 3T3L1-CRHR1 cells (lower panel) stimulated with 100 nM CRH or 50 µM forskolin (FSK). Data: mean ± SEM, n = 4.

A representative photograph is shown for each treatment. Scale bars, 50 µm.

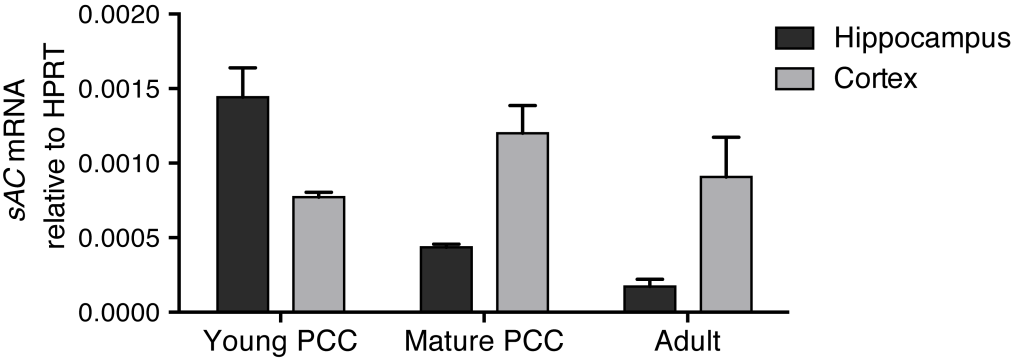

Supplementary Figure 3 l *sAC* expression in hippocampal and cortical primary cell cultures
Expression of *sAC* was assessed by RT-q-PCR in hippocampal or cortical neurons. RNA levels of CRHR1 were analyzed in young (DIV 5) and mature (DIV 12) primary cultures (PCC) and in the adult brain structures. *sAC* mRNA levels were normalized to *Hprt* (mean ± SEM, n = 3).

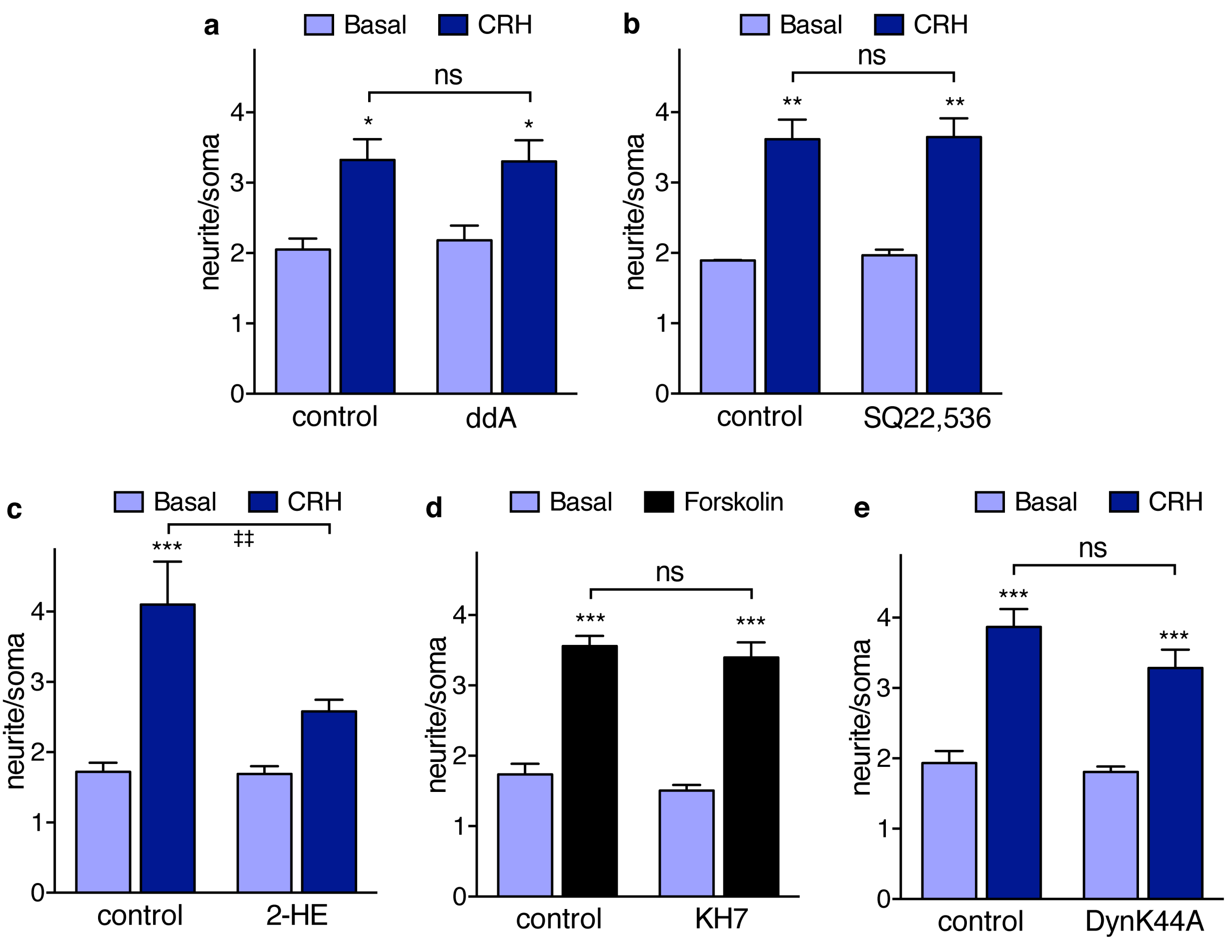

Supplementary Figure 4 I CRH-mediated neurite outgrowth depends on sAC activation

a-d, Neurite outgrowth was quantified in HT22-CRHR1 cells stimulated with 100 nM CRH (**a-c**) or 50 µM forskolin (**d**) in presence of vehicle (control), tmAC inhibitor (**a**, 50 µM ddA or **b**, 150 µM SQ22,536) or sAC inhibitor (**c**, 20 µM 2-HE or **d**, 7.5 µM KH7). Data: mean ± SEM, n = 3.

c, Neurite outgrowth was determined in HT22-CRHR1 cells transfected with pcDNA3 (control) or DynK44A 48 h before stimulation with 100 nM CRH. Data: mean ± SEM, n = 3.

*, $p < 0.05$, **, $p < 0.01$, ***, $p < 0.001$ respect to basal; ‡‡, $p<0.01$ between indicated treatments by one-way ANOVA followed by Tukey post test.

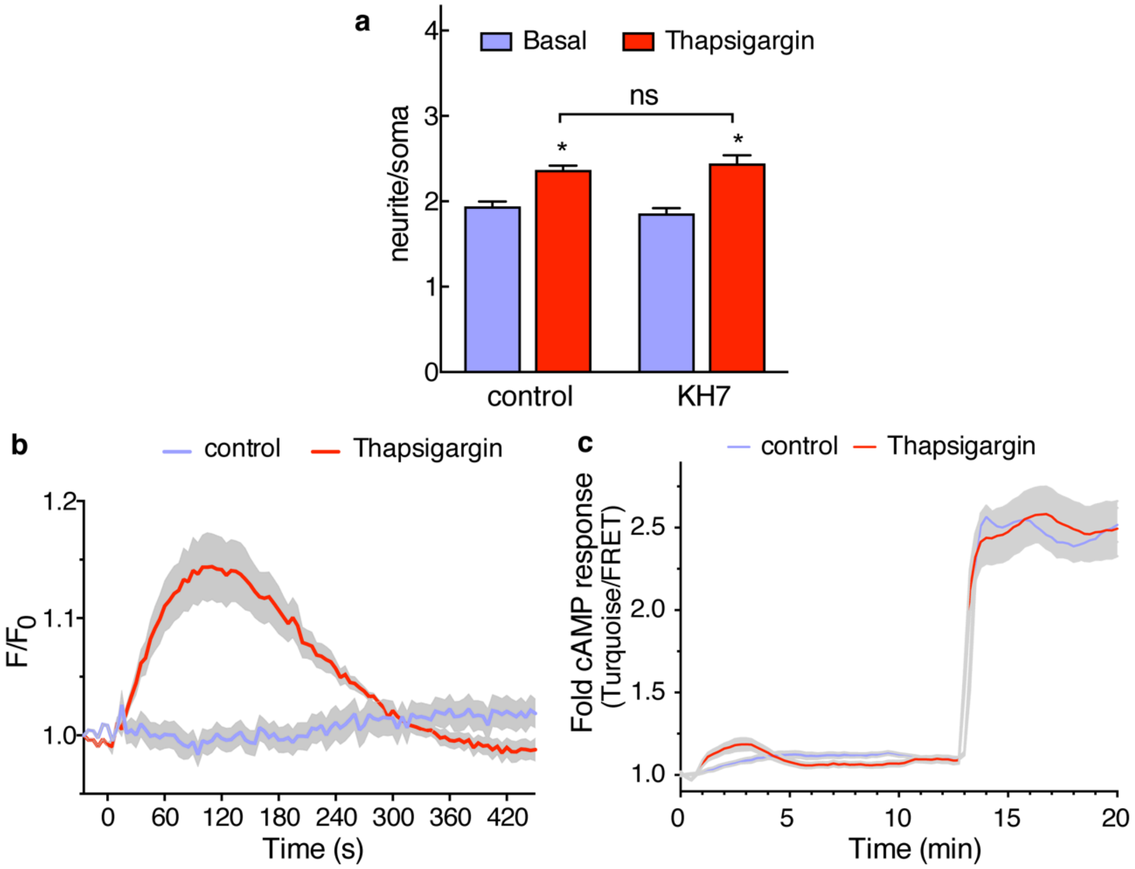

Supplementary Figure 5 I CRH-mediated neurite outgrowth depends on PKA activation

a, Neurite outgrowth was quantified in HT22-CRHR1 cells stimulated with 2 µM thapsigargin in presence of vehicle (control) or sAC inhibitor (7.5 µM KH7). Data: mean ± SEM, n = 3. *, $p < 0.05$ respect to basal.

b, Calcium response was determined in HT22-CRHR1 cells loaded with Fluo-4-AM, and stimulated at time 0 with vehicle or 2 µM thapsigargin. Data: mean ± SEM, n = 5.

c, cAMP levels were determined as FRET changes in HT22-CRHR1-Epac-S^{H187} cells stimulated with vehicle or thapsigargin in phenol red–free DMEM. Data: mean ± SEM, 15–17 cells.

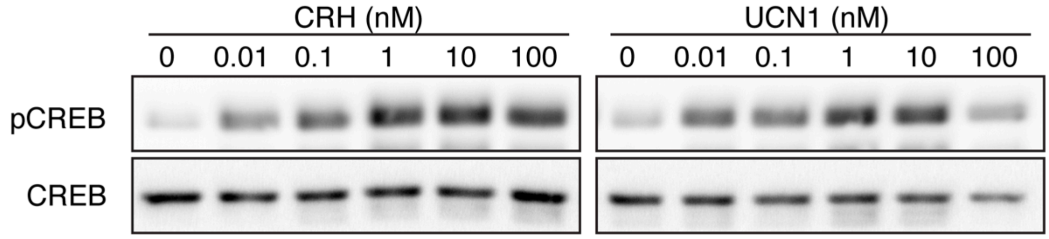

Supplementary Figure 6 I CREB activation by CRH and UCN1 is concentration-dependent
HT22-CRHR1 cells were stimulated for 40 min with the indicated concentrations of CRH or UCN1. Phosphorylated CREB (pCREB) and total CREB were determined by Western blot.

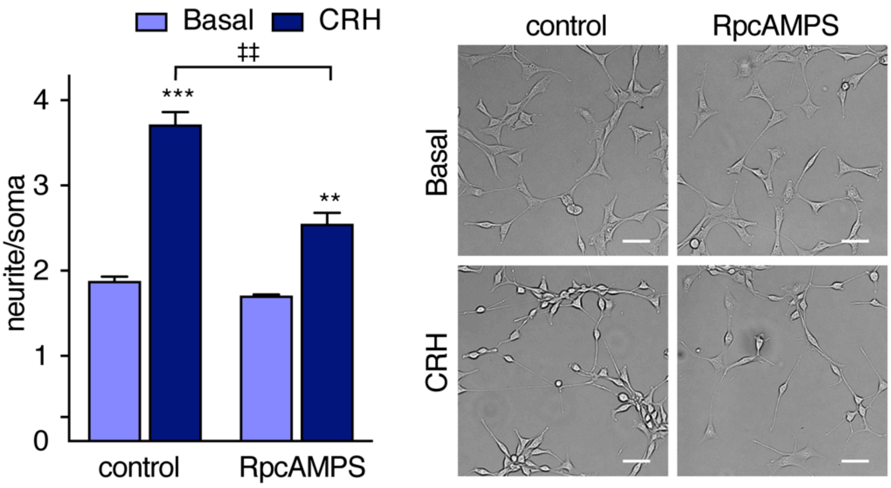

Supplementary Figure 7 I CRH-mediated neurite outgrowth depends on PKA activation
Neurite outgrowth was quantified in HT22-CRHR1 cells stimulated with 100 nM CRH in presence of vehicle (control) or PKA inhibitor (50 µM RpcAMPS). Data: mean ± SEM, n = 3. **, $p < 0.01$, ***, $p < 0.001$ respect to basal; ‡, $p < 0.05$ between indicated treatments by repeated measures one-way ANOVA followed by Tukey post test. A representative photograph is shown for each treatment. Scale bars, 50 µm.

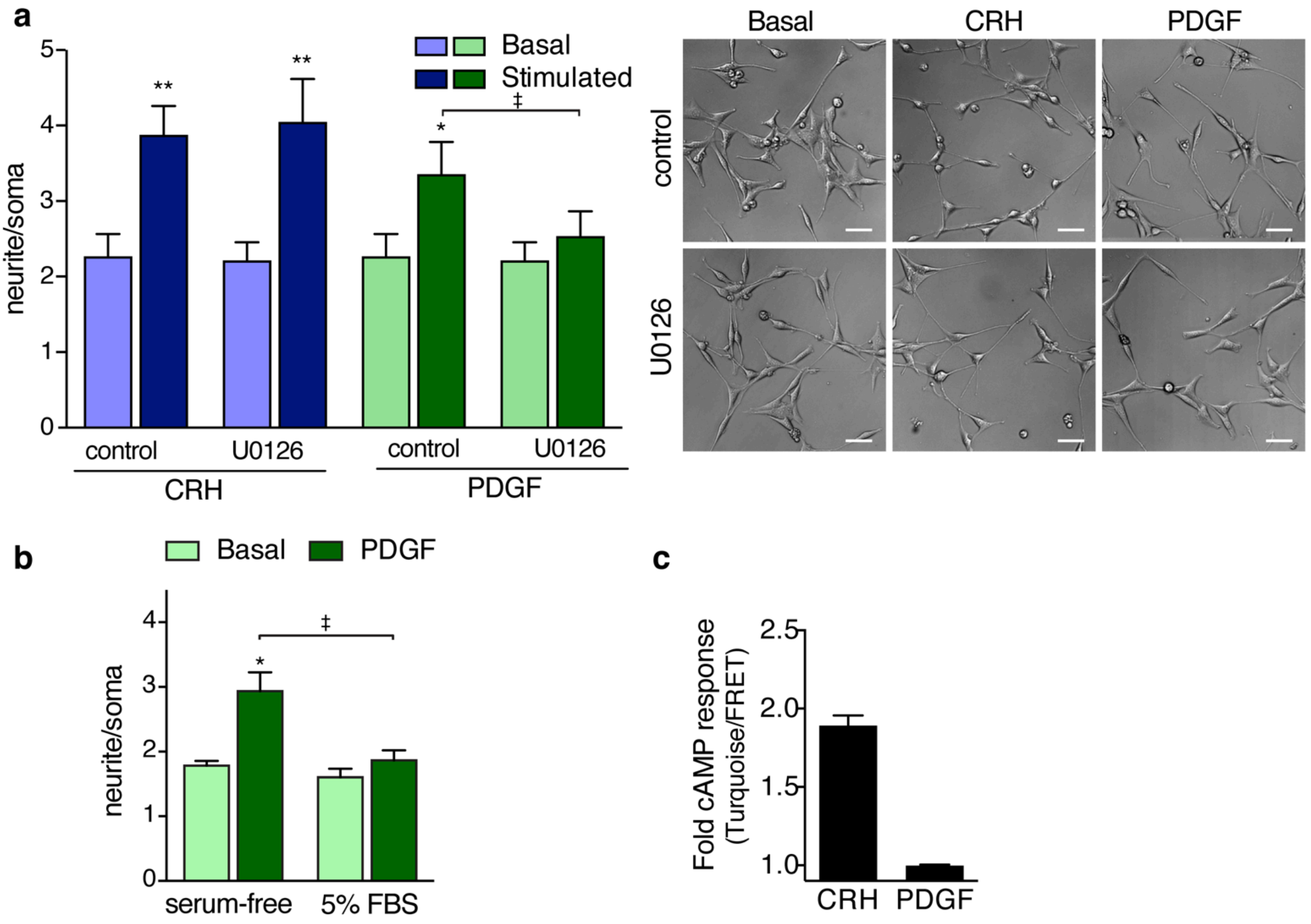

Supplementary Figure 8 | PDGF exerts a neuritogenic effect via ERK1/2 in HT22-CRHR1 cells

a, Neurite outgrowth was determined in HT22-CRHR1 cells stimulated with 100 nM CRH or 10 ng/ml PDGF in presence of vehicle (control), PKA-specific (10 µM H89), or MEK1/2-specific (10 µM U0126) inhibitors. Data: mean ± SEM (n = 3). ***, p < 0.001 respect to basal, ‡, p < 0.05 between indicated treatments by repeated measures one-way ANOVA followed by Tukey post test. A representative photograph is shown for each treatment. Scale bars, 50 µm.

b, Neurite outgrowth was quantified in HT22-CRHR1 cells stimulated with 10 ng/ml PDGF in serum-free media or in presence of 5% FBS. Data: mean ± SEM (n = 3). *, p<0.05 respect to basal, ‡, p < 0.05 between indicated treatments by repeated measures one-way ANOVA followed by Tukey post test.

c, cAMP levels were determined as FRET changes in HT22-CRHR1-Epac-S^{H187} cells stimulated with 100 nM CRH or 10 ng/ml PDGF in phenol red–free DMEM. Bars represent the maximum FRET change respect to the basal. Data: mean ± SEM, 20–25 cells from three independent experiments.

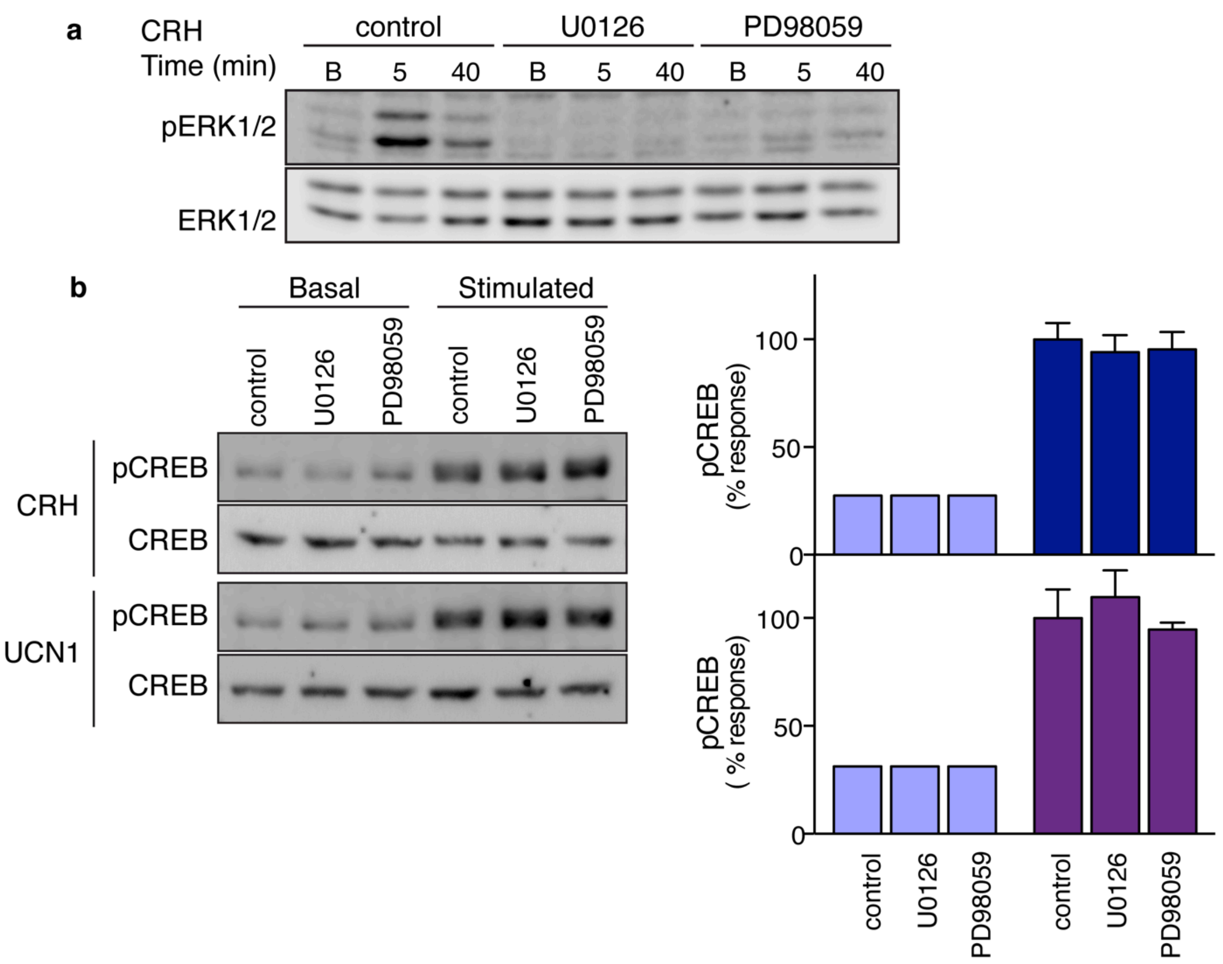

Supplementary Figure 9 I CREB activation does not depend on ERK1/2 phosphorylation

HT22-CRHR1 cells were stimulated with 100 nM CRH or UCN1 in presence of vehicle (control), or MEK1/2 inhibitors 10 µM U0126 or 10 µM PD98059. **a**, phosphorylated ERK1/2 (pERK1/2) and total ERK1/2 were determined by Western blot at the indicated time points. **b**, phosphorylated CREB (pCREB) and total CREB were determined by Western blot in cell lysates after 40-min of stimulation. Signals were relativized to total CREB and normalized to basal. Results are expressed as the percentage of pCREB in control conditions (mean ± SEM, n = 3).
